# Effective and scalable single-cell data alignment with non-linear canonical correlation analysis

**DOI:** 10.1093/nar/gkab1147

**Published:** 2021-12-06

**Authors:** Jialu Hu, Mengjie Chen, Xiang Zhou

**Affiliations:** Department of Biostatistics, University of Michigan, Ann Arbor, MI 48109, USA; Department of Human Genetics and Department of Medicine, University of Chicago, Chicago, IL 60637, USA; Department of Biostatistics, University of Michigan, Ann Arbor, MI 48109, USA; Center for Statistical Genetics, University of Michigan, Ann Arbor, MI 48109, USA

## Abstract

Data alignment is one of the first key steps in single cell analysis for integrating multiple datasets and performing joint analysis across studies. Data alignment is challenging in extremely large datasets, however, as the major of the current single cell data alignment methods are not computationally efficient. Here, we present VIPCCA, a computational framework based on non-linear canonical correlation analysis for effective and scalable single cell data alignment. VIPCCA leverages both deep learning for effective single cell data modeling and variational inference for scalable computation, thus enabling powerful data alignment across multiple samples, multiple data platforms, and multiple data types. VIPCCA is accurate for a range of alignment tasks including alignment between single cell RNAseq and ATACseq datasets and can easily accommodate millions of cells, thereby providing researchers unique opportunities to tackle challenges emerging from large-scale single-cell atlas.

## INTRODUCTION

Single cell sequencing (1–3) has been transformative in studies of gene regulation (4), cellular differentiation (5) and cellular diversity (6). As the technologies have been improved dramatically over recent years, the number of single cells assayed by each experiment has increased exponentially, along with a rapid growth and accumulation of datasets produced from large-scale studies (7). Consequently, a major computational challenge in current single cell studies is the standardization of measurements from multiple different samples or across different platforms and data types for effective integrative and comparative analyses. Such integrative analysis requires the development of single cell data alignment methods that can remove batch effects and account for technical noises across datasets (8).

Many single cell data alignment methods have been recently developed. The majority of them, with a few notable exceptions such as the recent iNMF (9), are targeted towards small-size and medium-size datasets. These existing methods can be summarized into four categories: (i) reference-based methods, such as Scmap-cluster (10) and scAlign (11), which align new inquiry datasets based on a well-annotated reference dataset; (ii) clustering-based methods, such as Harmony (12), DESC (13), which remove batch effects and align samples in an embedding space by iteratively optimizing a clustering objective function; (iii) matching-based methods, such as MNN (14) and Scanorama (15), which apply a mutually nearest neighbors strategy to identify overlapped cells across datasets and (iv) projection-based methods that use a statistical model to project individual cells from different datasets into a lower dimensional space, including Seurat (16,17) that applies canonical correlation analysis for projection, LIGER (18) that uses latent factors from non-negative matrix factorization for projection, and scVI (19,20) and others (21–23) that use variational techniques for projection.

Unfortunately, most existing alignment methods have intrinsic drawbacks that prevent them from successful applications to large-size datasets. Specifically, the alignment by reference-based methods will be limited by the reference data size and the pre-selected cell type annotations available in the reference, thus can lead to an increasing chance of missing new discoveries when data size increases. The matching-based methods like MNN use a round-trip walk strategy that requires generation of all pairwise alignments for datasets with more than two samples, which will be time-consuming for large sample sizes. The methods with sophisticated parametric models such as LIGER and scAlign or methods with complicated post-hoc data processing such as Seurat are also challenging to be scaled up to large-size datasets. The ZINB-based methods such as scVI may be less efficient in capturing complex expression features for multiple datasets. Although some existing recent methods (9) can be scaled up to large-size datasets, they still have potential to inaccurately align cells due to complicated parametric models. Therefore, it is in urgent need to develop effective alignment methods that are also computationally efficient.

Besides the urgent need of developing scalable alignment methods, another impeding issue of the current alignment methods is that their performance is often benchmarked and optimized using only single cell RNA sequencing (scRNA-seq) data. Consequently, most existing alignment methods are not ideal for integrating other single cell sequencing data types such as single cell assay for transposase-accessible chromatin using sequencing (scATAC-seq) (24). Furthermore, the returned results from existing alignment methods such as Seurat can only retain the true cell-cell relationships (or similarities), while they don’t represent gene expression levels which make them unsuitable for downstream analyses such as differential expression analysis or enrichment analysis (17).

To tackle these challenges, we propose a unified computational framework, VIPCCA, based on a non-linear probabilistic canonical correlation analysis, for effective and scalable single cell data alignment. VIPCCA leverages cutting-edge techniques from deep neural network for non-linear modeling of single cell data, thus allowing users to capture the complex biological structures from integration of multiple single-cell datasets across technologies, data types, conditions, and modalities. In addition, VIPCCA relies on variational inference for scalable computation, enabling efficient integration of large-scale single cell datasets with millions of cells. Importantly, VIPCCA can transform multi-modalities into lower dimensional space without any post-hoc data processing, a unique and desirable feature that is in direct contrast to existing alignment methods. We show through extensive real data analyses that the latent factors or variables in the shared lower dimensional space of VIPCCA can be used for clustering cell subpopulations, trajectory inference, and transfer learning across modalities, and that the aligned data recovered in the original data space by VIPCCA support effective downstream differential gene expression analysis.

## MATERIALS AND METHODS

### Model specification

We consider the analytic task of aligning *k* different single cell sequencing datasets into a common low-dimensional manifold. These *k* different datasets may represent different samples, studies, data types, sequencing techniques, and/or experimental conditions. We assume that the input data matrix from the *m*-th dataset, ${X^{( m )}}$ ($m\ = \ 1, \cdots ,k$), is of dimensionality ${n_m}$ by *p*, measuring a common set of *p* features in ${n_m}$ different cells. These feature measurements are in the form of gene expression levels when the sequencing technology is scRNA-seq, representing the read counts mapped to each gene in each cell; and in the form of gene-specific chromatin accessibility levels when the sequencing technology is scATAC-seq, representing a gene activity matrix that summarizes the ATAC-seq peak counts on each gene in each cell. Because our model requires the features to be common across datasets, we naturally use genes as features for both scRNA-seq and scATAC-seq in the present study. Here, we assume that the feature measurements have been properly normalized according to the sequencing technology (details in the next section). Our goal is to align these input data matrices together and project them onto a common low-dimensional latent space. To do so, we assume that the *p* feature measurements for each cell, regardless which study the cell comes from, is originated from a common low-dimensional space with dimensionality *d* (*d* < *p*). We denote the latent representation of ${X^{( m )}}$ as ${Z^{( m )}}$, which is an ${n_m}$ by *d* matrix of latent factors. We denote $X_i^{( m )}$ and $Z_i^{( m )}$ as the *i*th row-vector of ${X^{( m )}}$ and ${Z^{( m )}}$, respectively. We assume that the *p*-vector of features $X_i^{( m )}$is connected to the *d*-vector of latent factors $Z_i^{( m )}$ in the following form

(1) $$\begin{equation*}X_i^{\left( m \right)} = f\left( {Z_i^{\left( m \right)}\ ,{b^{\left( m \right)}}{\rm{|}}\theta } \right) + E_i^{\left( m \right)},\end{equation*}$$

where $f( \cdot )$ is a non-linear function defined with a set of parameters ${\rm{\theta }}$; ${b^{( m )}}$ is a dataset specific vector with a dimensionality ${l_b}$ for modeling dataset specific effects; and $E_i^{( m )}$ is a *p*-vector of residuals errors where each element independently and identically follows a normal distribution $\mathcal{N}( {0,{\sigma ^2}} )$. We use Gaussian to characterize the distribution of the residual errors as we directly use normalized data instead of count data as input. For the latent factors $Z_i^{( m )}$ in equation (1), we follow traditional factor analysis models and assume that each element of $Z_i^{( m )}$ follows a standard normal distribution

(2) $$\begin{equation*}Z_i^{\left( m \right)} \sim \mathcal{N}\left( {0,I} \right),\end{equation*}$$

with the covariance being a *d* by *d* diagonal matrix $I$. The assumption in equation (2) ensures that the latent variables from different datasets all reside on a common low-dimensional space.

In the above model defined in Equations (1 and 2), two terms are of particular importance: $f( \cdot )$ and ${b^{( m )}}$. The function$\ f( \cdot )$ is shared across all datasets and relates the latent variables $Z_i^{( m )}$ to $X_i^{( m )}$ through the same non-linear functional form defined by a common set of parameters ${\rm{\theta }}$. The parameter ${b^{( m )}}$, on the other hand, is dataset specific and effectively determines how the latent variables $Z_i^{( m )}$ are related to $X_i^{( m )}$ differently in different datasets. When $f( \cdot )$ is a linear function and when ${b^{( m )}}$ is absent, the model defined in Equations (1 and 2) reduces to the standard probabilistic CCA model, which expresses $X_i^{( m )}$as a weighted function of $Z_i^{( m )}$ with weights being stored in a factor loading matrix (25). Consequently, the non-linear function$\ f( \cdot )$ shared across datasets and the dataset specific vectors ${b^{( m )}}$ all together generalize the standard probabilistic CCA model towards modeling both non-linear and data specific effects that are key for ensuring effective data integration. The detailed relationship of our method with the probabilistic CCA and other statistical models is provided in the Supplementary Text. Importantly, our method defined in Equations (1 and 2) is also a data generative in nature and describes how different datasets are originated from a common shared latent space along with dataset specific features.

Technically, we use deep neural network to construct the non-linear functional form of $f( \cdot )$. The detailed construction is provided in the Supplementary Text. Briefly, the neural network takes input of the *d*-dimensional vector $Z_i^{( m )}$ and the ${l_b}$-dimensional vector ${b^{( m )}}$, and outputs a *p*-vector of outcomes through several intermediate neural network layers. We set the dimensionality $d$ and ${l_b}$ to the default value of 16 throughout the study and we randomly generate each element of ${b^{( m )}}$ from a discrete uniform distribution *U*(0, 10). We construct the intermediate layers in the neural network to be fully connected with each other through rectified linear unit (ReLU) functions. Note that the alignment results are robust with respect to the different ${b^{( m )}}$ generated from different random seeds, with respect to the dimensionality of ${b^{( m )}}$, and with respect to the dimensionality of the bottle layer (Supplementary Figures S15-17), thanks to the flexibility of the deep neural network modeling framework. We also include a batch normalization layer inserted before each ReLU activation layer to accelerate inference and include a dropout layer interested after each ReLU activation layer to avoid model overfitting.

Our goal is to obtain the posterior estimates of the latent variables $Z_i^{( m )}$ based the feature matrices $X_i^{( m )}$. Obtaining these posterior estimates is challenging because of the non-linear function $f( \cdot )$. In particular, the posterior estimates of $Z_i^{( m )}$ based on the likelihood defined in Equations (1 and 2) cannot be computed analytically when $f( \cdot )$ is a non-linear function. To enable effective and scalable inference, we develop a variational approximation algorithm (details in the Supplementary Text). Briefly, we introduce a relatively simple variational distribution ${\rm{q}}( {Z_i^{( m )}} )$ to approximate the complex posterior distribution $p( {Z_i^{( m )}|X_i^{( m )},\ {b^{( m )}}} )$. The variational distribution is assumed in the form of a multivariate normal distribution $MVN( {{\rm{\mu }}_{\rm{i}}^{( m )},{\rm{diag}}( {{\rm{\sigma }}_{\rm{i}}^{2( {\rm{m}} )}} )} )$, with a *d*-dimensional mean vector ${\rm{\mu }}_{\rm{i}}^{( m )}$ and a diagonal covariance matrix consists of a *d*-dimensional variance vector of ${\rm{\sigma }}_{\rm{i}}^{2( {\rm{m}} )}$. Because the posterior distribution for $Z_i^{( m )}$ is a non-linear function of the observed expression data $X_i^{( m )}$, we also use a non-linear function constructed by a similar form of the neural network to characterize the mean and variance parameters in the variational distribution ${\rm{q}}( {Z_i^{( m )}} )$. With the variational distribution, we minimize the Kullback-Leibler (KL) divergence between ${\rm{q}}( {Z_i^{( m )}} )$ and $p( {Z_i^{( m )}|X_i^{( m )},\ {b^{( m )}}} )$ to obtain ${\rm{\mu }}_{\rm{i}}^{( m )}$ to serve as the estimates of $Z_i^{( m )}$. In the variational inference, we rely on an optimization technique based on mini-batch gradient descent to ensure scalable computation. With mini-batch gradient descent, we iterate through all single-cell datasets repeatedly with a default of 100 epochs. We update the parameters in accordance with the gradient of error with respect to a subset of the datasets with a batch size set to be 256. In the algorithm, we rely on early-stop settings and take advantage of the reduce learning rate strategies. The overall computational complex is $O( {n{p^5}} )$, with memory usage $O( {{p^2}} )$. Here, $n$ and $p$ are the total number of cells and selected genes, respectively.

One key feature of VIPCCA that differs from many existing integration methods is its ability to remove dataset specific batch effects and obtain normalized gene expression data. Specifically, we first obtain the estimated dataset-specific embedding in the low dimensional space (i.e. $\hat{Z}_i^{( m )}$) and then paired it with the dataset-specific annotation (i.e. ${b^{( m )}}$) to recover the dataset-specific gene expression through the non-linear function $f( \cdot )$. With the parameter estimates $\hat{\theta }$, the recovered gene expression data $\hat{X}_i^{( m )}$ is in the form of $\hat{X}_i^{( m )}{\rm{\ }} = \ f(\hat{Z}_i^{( m )},{b^{( 1 )}}|\hat{\theta })$.

We refer to the above method as the Variational Inference assisted Probabilistic Canonical Correlation Analysis (VIPCCA) because of its close relationship to CCA and the underlying variational inference algorithm. VIPCCA is implemented in a python package, vipcca (≥0.2.6), freely available at https://github.com/jhu99/vipcca.

### Compared methods

We compared VIPCCA with seven existing integration methods using default software settings. These methods include (i) DESC (v0.1.6.1), where we followed tutorial and used the functions normalize_per_cell, log1p in the scanpy package for data preprocessing; (ii) harmony (v1.0), where we followed software recommendations (12) and used NormalizeData, ScaleData, RunPCA with npcs = 16 in Seurat for data preprocessing; (iii) LIGER (v0.4.2), where we followed software recommendations and used NormalizeData, ScaleData with scale.factor = 1e6, do.center = F in Seurat for data preprocessing; (iv) MNN (Batchlor/mnn_correct v1.2.4), where we followed software recommendations to use the preprocessed data from Seurat as input and the mnnCorrect function with *k* = 16; (v) ScAlign (v1.2.0), where we followed tutorial and used the functions NormalizeData, ScaleData with scale.factor = 1e6, do.scale = T and do.center = T in Seurat for data preprocessing; (vi) Scanorama (v1.5.0), where we followed software recommendation and used the functions correct_scanpy with dimred = 16; (vii) scVI (v0.5.0), where we followed tutorial and used the count data as input and (viii) Seurat (v3.1.0.9007), where we followed tutorial and used function NormalizeData with scale.factor = 1e6 for data preprocessing.

### Real datasets

We applied our method and other existing integration methods to analyze a total of 17 published single cell sequencing datasets through five applications.

In the first data application, we obtained eight scRNA-seq datasets on human pancreatic islets that span 27 donors and five scRNA-seq technologies (CelSeq, CelSeq2, FluidigmC1, SMART-seq2, InDrops). These data are available either at the NCBI Gene Express Omnibus (GEO) website (ID GSE84133 (26), GSE86469 (27), GSE81076 (28) and GSE85241 (29)) or the ArrayExpress repository (ID E-MTAB-5061 (30)). We obtained processed data directly from the SeuratData package. The raw count data contains a total of 34 363 genes measured on 14 890 cells from 13 cell types that include acinar (*n* = 1864), activated stellate (474), alpha (4615), beta (3679), delta (1013), ductal (1954), endothelial (296), epsilon (30), gamma (625), macrophage (79), mast (56), quiescent stellate (180) and schwann (25).

In the second data application, we obtained three published datasets (31) from the 10X Genomics single-cell portal: one on 2885 293T cells (https://support.10xgenomics.com/single-cell-gene-expression/datasets/1.1.0/293t); one on 3285 Jurkat cells (https://support.10xgenomics.com/single-cell- gene-expression/datasets/1.1.0/jurkat); and one on a 50:50 mixture of 1605 293T and 1783 Jurkat cells (https://support.10xgenomics.com/single-cell-gene-expression/datasets/1.1.0/jurkat:293t_50:50). All data are measured on a common set of 32 738 genes. The cell type labels of the 50:50 mixed samples were determined in the original publication based on the expression of cell type-specific markers CD3D (Jurkat) and XIST (293T).

In the third data application, we obtained a scRNA-seq data and a scATAC-seq data. The scRNA-seq data consists of gene expression measurements on 33,538 genes in 11 769 cells. The scATAC-seq data consists of 89 796 open chromatin peaks measured on 8728 nuclei. Both these data were produced by 10X Genomics Chromium system and were on PBMCs. We downloaded the processed data from 10X genomics website (cell-expression/3.0.0/pbmc_10k_v3 and cell-atac/1.0.1/atac_v1_pbmc_10k). We obtained 13 cell types in the scRNA-seq data using the standard workflow in Seurat (https://www.dropbox.com/s/3f3p5nxrn5b3y4y/pbmc_10k_v3.rds?dl=1). The 13 cell types include 460 B cell progenitor, 2992 CD14 + Monocytes, 328 CD16 + Monocytes, 1596 CD4 Memory, 1047 CD4 Naïve, 383 CD8 effector, 337 CD8 Naïve, 74 Dendritic cell, 592 Double negative T cell, 544 NK cell, 68 pDC, 52 Plateletes and 599 pre-B cell. In addition to the two single cell sequencing data, we also obtained a bulk ATAC-seq data on a subset of human immune cell types from the UCSC Genome Browser (https://s3-us-west-1.amazonaws.com/chang-public-data/2016_NatGen_ATAC-AML/hub.txt). For the scATAC-seq data, we filtered out cells that have with fewer than 5000 total peak counts to focus on a final set of 7,866 cells for analysis.

In the fourth data application, we obtained two large datasets: one scRNA-seq dataset (32) (http://dropviz.org) measured on 861 851 cells residing in nine regions of the mouse brain and one single nuclei sequencing dataset (33) (GSE110823) measured on 156 049 single nuclei in both mouse brain and spinal cord at two developmental stages (p2 and p11). The two data were profiled with Drop-seq and SPLiT-seq, respectively. The nine brain regions in the scRNA-seq data include cerebellum, entopeduncular, frontal cortex, globus pallidus, hippocampus, posterior cortex, striatum, substantia nigra and thalamus. We obtained the cell type annotations from the original publications (http://dropviz.org/ and https://www.ncbi.nlm.nih.gov/geo/query/acc.cgi?acc=GSE110823 with code at https://gist.github.com/Alex-Rosenberg/5ee8b14ea580144facad9c2b87cebf10). A total of 22 cell types and 187 subtypes were reported in the scRNA-seq dataset, and 73 cell types were reported in the SPLiT-seq dataset. The seven major cell types in both datasets are astrocytes, endothelial, fibroblast-like, microglia/macrophage, mural, oligodendrocytes, and polydendrocytes. For simplicity, we merged these 73 sub-celltypes into several main cell types following (34).

In the fifth data application, we obtained two scRNA-seq datasets on male and female human embryos: one normalized count data (TPM) of 2621 cells measured on 24 153 genes (GSE86146); one normalized count data (FPKM) of 328 cells measured on 23,394 genes (GSE63818). After filtering away somatic cells with the annotation data in the original publication, we used only 666 female (5–26W) 649 male (4–25W) PGCs in the first dataset; and 83 female (4–17W) 141 male (4–19W) PGCs in the second dataset. The annotation data can be freely accessible at https://zenodo.org/record/1443566/files/real/gold/germline-human-female-weeks_li.rds?download=1, https://zenodo.org/record/1443566/files/real/gold/germline-human-male-weeks_li.rds?download=1, https://zenodo.org/record/1443566/files/real/gold/germline-human-female_guo.rds?download=1, and https://zenodo.org/record/1443566/files/real/gold/germline-human-male_guo.rds?download=1.

### Data processing

For the scRNA-seq data, we used anndata (v0.6.22.post1) and scanpy (v1.4.4.post1) to normalize the expression count data in each data application following scanpy's tutorial. The only exception is the fifth application, which has already been normalized in the original study. For scanpy normalization, for each individual in turn, we first divided the gene count by the total read depth and computed counts per million mapped reads (cpm). We then performed log transformation on the resulting cpm values. In the log transformation, we added a pseudo count of 1 to avoid taking logarithm of zeros. The normalization procedure is achieved by using the function pp.normalize_total, with target_sum = 1e6 and the pp.log1p option. In the first, second and fourth applications, we first selected the top 2000 highly variables genes (HVGs) in each dataset separately, using the function pp.highly_variable_genes in scanpy with the option flavor = ‘Seurat’. We then obtained the 2000 HVGs among them that appeared in the largest number of batches to serve as the final set of features for all integration methods. In the third data application, by following (17), we obtained 3000 HVGs in the scRNA-seq data and focused on these genes for analysis. In the fifth data application, we used 2200 HVGs for male, 2918 for female PGCs, which were obtained from common genes of the two sets of HVGs used in (35,36).

For the scATAC-seq data, we examined one gene at a time and obtained a gene activity measurement by summing all peak counts within the genomic region between 2 kb upstream of the transcription starting site and the transcript ending site based on the reference human genome annotation file (ftp://ftp.ensembl.org/pub/grch37/release-84/gtf/homo_sapiens/Homo_sapiens.GRCh37.82.gtf.gz). We retained genes that are among the 3000 HVGs selected in scRNA-seq data and with non-zero activity measurements in at least one cell and we removed cells with less than 5,000 peaks. We focused on a final list of 2174 genes on 7866 cells for analysis. In the stage of label transferring, we calculated a prediction score for each scATAC-seq cell.

In each data application, we followed the integration workflow of each compared method for analysis. For the second application, LIGER failed on the original set of 2000 HVGs. Therefore, we had to use an alternative set of ∼1600 HVGs for LIGER. These genes were common HVGs shared by HVGs of the three input datasets, obtained by using similar variable selection function FindVariableFeatures in the Seurat package with method = ‘vst’ and nfeatures = 2000. For the third data applications, we carried out Seurat analysis exactly follow its tutorial on the data (https://satijalab.org/seurat/v3.0/atacseq_integration_vignette.html).

We did not scale and center the gene expression measurements in VIPCCA because our neural network requires nonnegative values. The p-vector outcome (i.e. non-negative) of VIPCCA was calculated by adding two layers with softplus and hard_sigmoid activation functions.

To avoid causing unpredictable loss of algorithm performance, we preprocessed the count data by following the tutorial of each package for preprocessing example datasets. Hence, there are some slight differences in the data processing of other algorithms: (i) DESC does not scale and center the data after the log1p transformation; (ii) scanorama and scVI take the count data as input; (iii) harmony, MNN, scAlign and Seurat scale and center the data after the log1p transformation; (iv) LIGER scale but do not center the data after the log1p transformation.

### Evaluation metric for single-cell integration

We compared the performance of different integration methods using three evaluation criteria: kBET acceptance rate (37), mixing metric (17) and adjusted rand index (ARI). Both kBET and mixing metric assess how well mixed the integrated data are, while ARI assesses how well cell types are inferred. All these metrics are computed based on the aligned low dimensional space following standard practice in the field (17,34). Because kBET test is computationally slow and requires a huge amount of physical memory, we followed the recommendation of kBET user manual (https://github.com/theislab/kBET) and used a subsampling strategy on the fourth dataset that is of particular large-scale. Since ARI requires both predicted clustering labels and the true cell type label, we performed clustering with the FindClusters function in Seurat, which is based on the Louvain algorithm (38), to perform clustering on all cells in the aligned reduced dimensional space. The true cell type labels were obtained from the original publication of each dataset. Following (34), we performed kBET test (v0.99.6) for each cell type over a range of neighborhood sizes that cover 5–25% of the sample size, with 100 replicates for each neighborhood size. In the last large-scale data application, kBET tests were examined on 4,000 samples randomly selected from each of the two datasets to ensure computational efficiency. We computed mixing metric (17) using the function LocalStruct in Seurat V3, where we set *k* = 5, k.max = 300 as recommended in (17). Finally, we used adjusted rand index (ARI) to measure the accuracy of cell type clustering following (13). A high value of kBET test statistics, mixing metric, or ARI indicates better performance.

### Differential gene expression analysis

To test whether the batch-effects were effectively removed without over-correction or less-correction, we performed gene expression analysis on recovered expression data in three scenarios: (i) differentially expressed genes (DEGs) between two batches within one cell type; (ii) DEGs between two cell types within one batch; (iii) DEGs between two cell types of two different batches. To do so, we used FindMarkers with test.use = ‘wilcox’ in Seurat for identifying differentially expressed genes on the same set of common variables across all compared algorithms. So, a Wilcoxon rank-sum test was applied onto normalized expression data of each integration algorithm on a same set of 2000 highly variable genes (13,17), which were selected by using scanpy.pp.highly_variable_genes function with flavor = ‘Seurat’. The differentially expressed genes were identified by using FindMarkers in the Seurat package (16) with an FDR control of 0.05 (Bonferroni correction).

In the first data application, we tested DEGs between celseq and celseq2 within each of four cell types (acinar, alpha, beta, ductal) in the first scenario. We selected the two datasets and the four cell types because they have relatively larger number of cells so that each compared group in the two datasets has at least 50 cells involved. We tested DEGs between alpha and beta within celseq in the second scenario, and DEGs between alpha from celseq and beta from celseq2 in the third scenario. In the second data application, we tested DEGs between the first and the third datasets within 293T, and DEGs between the second and the third datasets within Jurkat in the first scenario. We tested DEGs between 293T and Jurkat within the third dataset in the second scenario, and DEGs between 293T from the first dataset and Jurkat from the third dataset in the third scenario. Jaccard index was used to quantify the similarity between the top100 DE genes detected in Scenario 2 and Scenario 3. The top 100 DE genes were obtained by sorting DE genes by their p_value_adj and –abs(avg_log2FC) in increasing order.

### Trajectory inference

We performed trajectory inferences using Slingshot (v2.0.0) (39) on female and male human primordial germline cells separately. The inferred pseudotime would reflect the cell state of PGCs in the development.

### Reference-based cell type assignment for scATAC-seq and its performance evaluation

For VIPCCA, we performed reference-based cell type assignment in the scATAC-seq data using the cell types annotated on the scRNA-seq data. Specifically, after VIPCCA integration, we constructed a neighborhood graph on the cell embeddings in the reduced dimensional space using the pp.neighbors in the scanpy package with n_neighbors = 50. For each scATAC-seq cell in turn, we then used a scoring function to calculate a confidence value for each cell type and assigned the cell type with the maximal score to the scATAC-seq cell as its cell type. The confidence score is calculated in the following form for the $i$th scATAC-seq cell and each celltype $c$:

$$\begin{equation*}{S_{ic}} = {\Sigma _{j \in {N_{ic}}}}\ {e^{ - \left( {\frac{{dist{{\left( {i,j} \right)}^2}}}{{2{w_c}}}} \right)}},\end{equation*}$$

where ${N_{ic}}$ represents the set of scRNA-seq neighbors of ${v_i}$ that are of cell type *c*; $dist( \cdot )$ returns the Euclidean distance measured on the latent space between the $i$-th cell in scATAC-seq and its $j$th neighbor cell in scRNA-seq; and the weight ${w_c}$ is predefined for the cell type *c* according to its global celltype proportion. Those query ones without any scRNA-seq neighbor, which are not well mixed with scRNA-seq cells, are assigned ‘unknown’.

We validated the cell type assignment in the scATAC-seq data by examining the scATAC-seq peaks in marker genes. To do so, we first used sinto (v0.7.1) to extract the scATAC-seq binary sequence/alignment map (BAM) file for each cell type according to the barcode of the scATAC-seq cells. We sorted and indexed each bam file using samtools (v1.7). We calculated the RPKM normalized read coverage tracks (bigwig format) on autosomes using bamCoverage in the deepTools (v3.4.3) with binSize = 1 and the RPKM normalization option. We then plotted the accessibility tracks of eight known cell type marker genes using CoveragePlot in Signac (v1.0.0). The marker genes include GNLY (marker of NK cells), MS4A1 (marker of pre-B and B cell progenitor), BCL11B (marker of CD4 Naïve, CD8 effector and CD8 Naïve cells), LYZ (marker of CD14 + monocytes, pDC), CD8A (marker of CD8 effector and CD8 Naïve cells), CD3E (marker of CD4N, CD8E, NK and CD8N), CD4 (marker of CD4N, CD4M, CD14M, CD16), HLA-DRA (marker of CD16 and CD14M) (17,40,41). We further calculated the average read coverage score for each marker gene and its flanking regions (10k upstream and 2k downstream) in assigned cell types in scATAC-seq using multiBigwigSummary in the deepTools based on the bigwig files. Besides examining the chromatin accessibility of marker genes in the assigned cell types, we also created pseudo-bulk ATAC-seq profile by pooling cells of each assigned cell type. We then compared the pseudo-bulk ATAC-seq data with the bulk ATAC-seq data of human immune cell types (B cells, NK cells, CD8+ T cells, CD4+ T cells, Monocytes) obtained from (https://s3-us-west-1.amazonaws.com/chang-public-data/2016_NatGen_ATAC-AML/hub.txt). In both pseudo-bulk and bulk ATAC-seq data, we computed the average score in equally sized bins (bin size = 10k bases) that consecutively covers the entire genome using multiBigwigSummary with the mode of bins. We then computed Pearson correlation for the global pattern of chromatin accessibility between pseudo-bulk and bulk ATAC-seq data across immune cell types (42).

## RESULTS

### VIPCCA method overview

**Figure 1.**
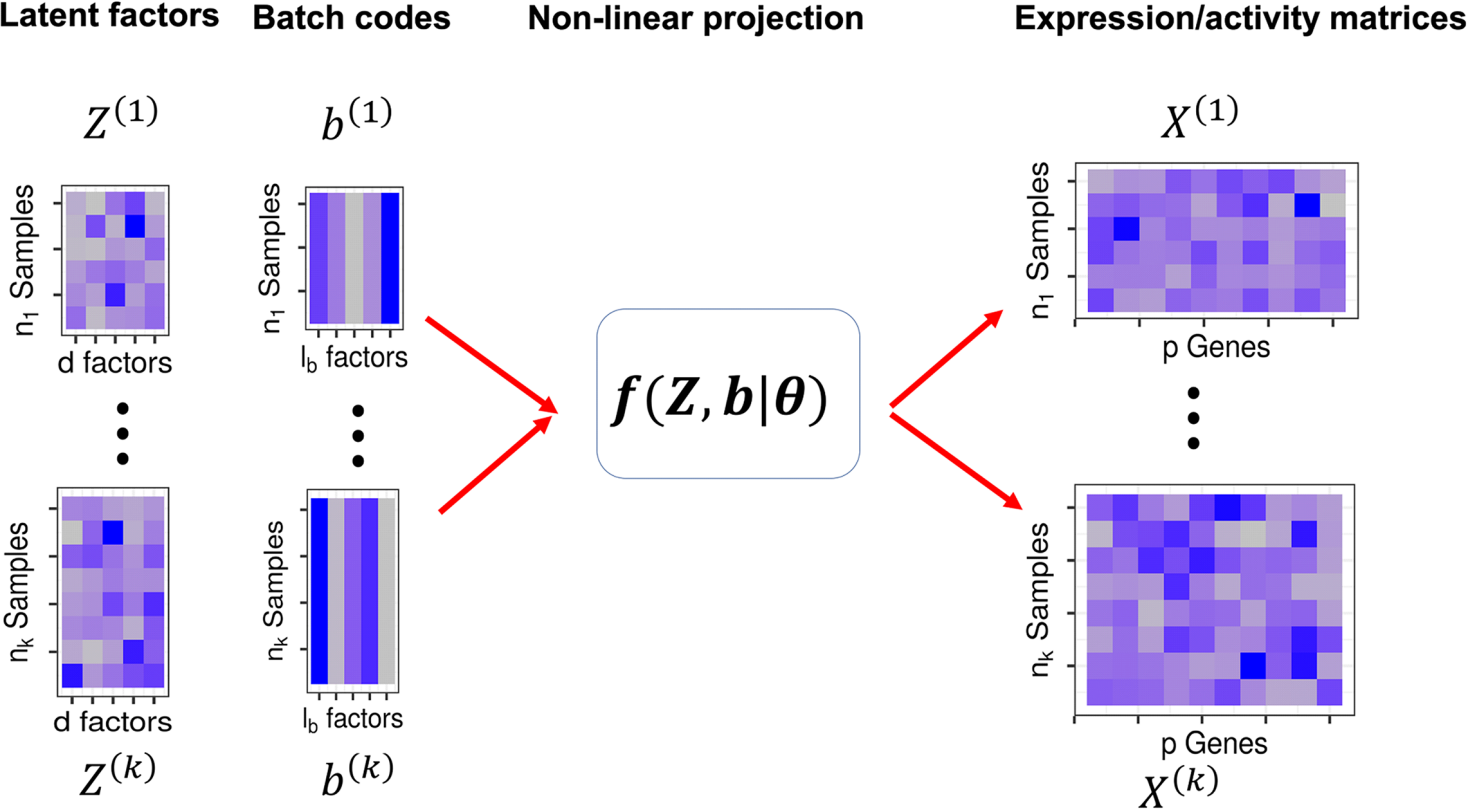
Method schematic for VIPCCA. VIPCCA takes an input from a normalized expression vector of $X_i^{( m )}$ from the $i$th cell in the $m$th single cell dataset and expresses it as a summation of two terms: a non-linear function term $f(\ \cdot |\theta )$ and a residual error term. The non-linear term is a function of a cell type specific latent factor $Z_i^{( m )}$ for the $i$th cell and a dataset-specific annotation code ${b^{( m )}}$ for the $m$th dataset. The non-linear function $f( \cdot |\theta )$ projects the latent factor into the original space with a non-linearity feature to flexibly capture the complex data structure across single cell datasets. The dataset-specific annotation code ${b^{( m )}}$ is introduced to model the dataset-specific variation that exists due to distinct experimental conditions, platforms, and technologies across datasets. VIPCCA infers the latent factors $Z_i^{( m )}$ and recovers the aligned expression values in the original space through a scalable variation inference algorithm.

A brief method schematic is provided in Figure 1 and technical details are provided in the Supplementary Text. Briefly, we consider the problem of aligning gene expression measurements (e.g. from scRNA-seq) and/or gene activity measurements (e.g. from scATAC-seq) obtained from multiple single-cell datasets and project them onto a common low dimensional space. To do so, we incorporate non-linearity into the commonly used canonical correlation analysis (CCA) and express the gene expression and/or gene activity matrix from each dataset as a non-linear function of the common latent factors. The non-linear function is constructed using a deep neural network with several interconnected layers. A unique feature of our method is its ability to incorporate dataset notational inputs into the neural network to represent dataset specific components of gene expression, thus allowing for flexible and accurate gene expression modeling across multiple scRNA-seq studies. Unlike the previous adaption of CCA for scRNA-seq alignment (16,17), our method is fully based on a data generative model, does not require any *post hoc* data processing or normalization, is applicable for a wide variety of alignment tasks coupled with various downstream analyses. To accompany our model, we develop a variational approximation algorithm for inference, which, when further paired with a mini-batch based stochastic gradient descent procedure, is highly computationally scalable. Because of the close relationship of our method to the probabilistic version of CCA, we refer to our method as the Variational Inference assisted non-linear Probabilistic CCA (VIPCCA). VIPCCA is freely available at https://github.com/jhu99/vipcca.

We examine the effectiveness of our method and compare it with seven existing alignment approaches through five real data applications. The compared approaches include DESC, Harmony, LIGER, MNN, scAlign, Scanorama, scVI and Seurat V3 (17) (details in Materials and Methods). The variety of real data applications we examined here aim to cover a wide range of integration tasks encountered in single cell data analysis.

### Cross-sample integration by VIPCCA leads to reliable and robust downstream analysis

Our first application focuses on integrating a collection of 34 363 genes measured on 14 890 cells from eight scRNA-seq datasets on human pancreatic islets that span 27 samples and five technologies (26–30) (Supplementary Figure S1). Before integration, the eight datasets were coupled with batch effects and technical biases (17). The challenge of the task here is thus to remove nuisance factors such as batch effects through data integration, while preserving the true expression signals for various downstream applications.

**Figure 2.**
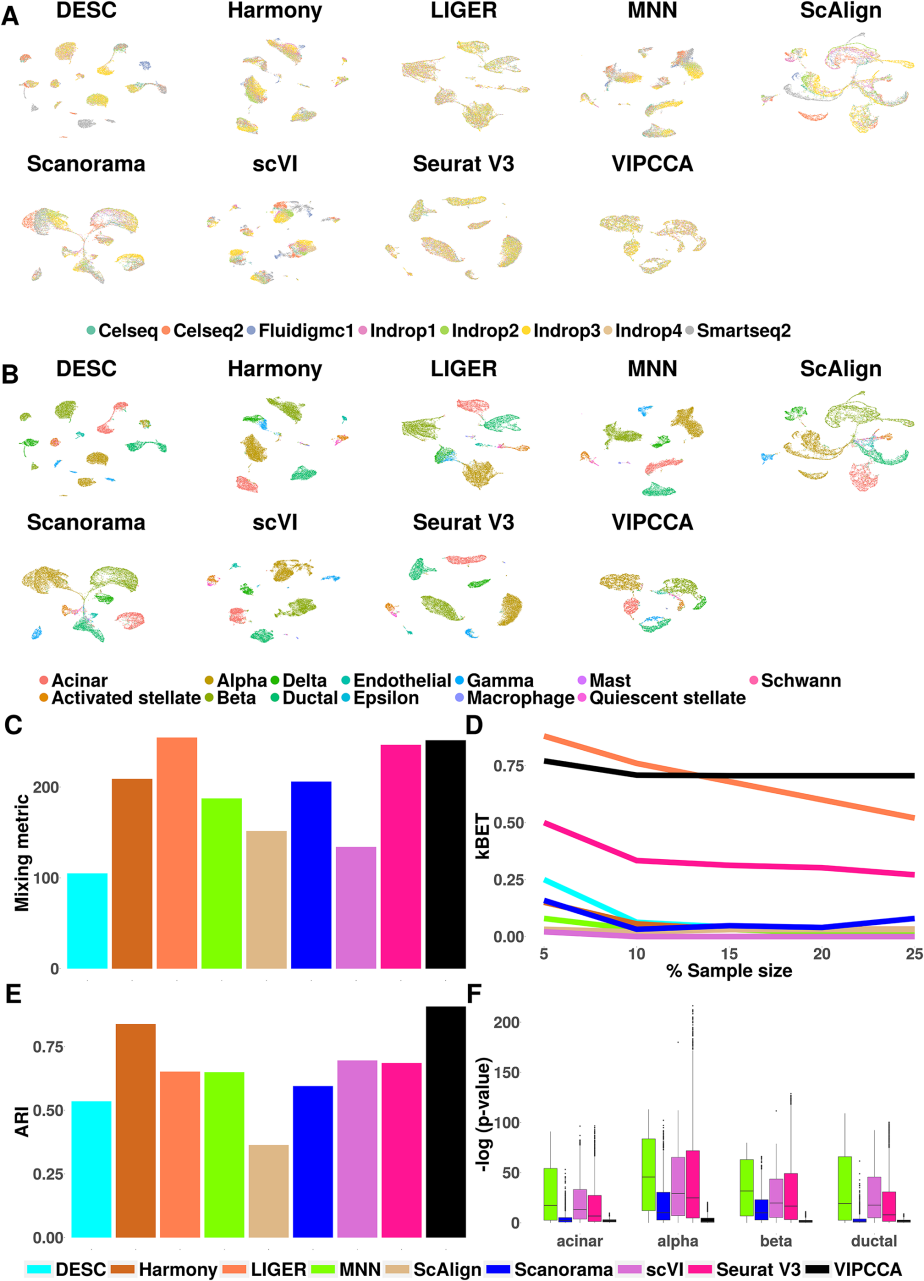
Integration of eight scRNA-seq datasets on human pancreatic islets. The examined datasets span 27 samples, five technologies, and four laboratories. The examined integration methods include DESC, Harmony, LIGER, MNN, scAlign, Scanorama, scVI, Seurat V3 and VIPCCA. UMAPs are used for visualizing integration results by either technologies (**A**) or cell types (**B**). The overall integration quality is measured by four metrics: mixing metric (**C**), kBET acceptance rate (**D**), adjusted rand index (ARI) (**E**) and differential expression analysis (**F**).

We first visualized the integrated data using UMAP (43) (Figure 2A, B). UMAP visualization suggests that cells from all eight datasets are well mixed after we apply the integration methods VIPCCA, LIGER, or Seurat, more so than the other six methods. In addition, cells in the integrated data obtained by VIPCCA, Harmony, or Seurat can be easily distinguished into different cell types, including three major types (delta, activated stellate, endothelial), more so than the other six methods. We further evaluated method performance using three metrics: the kBET acceptance rate (37), the mixing metric (17), and the adjusted rand index (ARI). Both the kBET acceptance rate and the mixing metric assess how well the cells are mixed after integration, while ARI assesses how well the cells can be clustered correctly into the 13 known cell types (Materials and Methods). Quantification supports the superior performance of VIPCCA, LIGER, Seurat in terms of mixing: their mixing metric scores are 251.5, 254.5 and 246.5, respectively, as compared to 105.0–206.0 for the rest (Figure 2C); VIPCCA and LIGER also achieve the highest kBET acceptance rates across cell types, which are followed by Seurat, and further followed by the rest (Figure 2D, Supplementary Figure S2). In terms of clustering, VIPCCA achieves the best performance (ARI = 0.91), followed by Harmony (0.84) and then the others (range from 0.36 to 0.70) (Figure 2E).

A key benefit of VIPCCA is its ability to generate normalized gene expression data that are free of batch effects for downstream analysis. Only three other methods (MNN, Scanorama, and Seurat) are capable of such gene expression recovery. Here, we followed (13,44) and evaluated the gene expression recovery accuracy of these four methods through differential expression (DE) analyses. Specifically, we first performed DE analysis on cells of the same cell type (either acinar, alpha, beta, or ductal) between two datasets (details in Materials and Methods). Intuitively, if the recovered data are free of batch effects, then we would not expect to observe strong DE evidence from such analysis. Indeed, the median −log_10_*P*-value across genes from DE analysis on VIPCCA recovered data is only 1.4, several folds lower than that of Scanorama (3.7), Seurat (12.5), scVI (18.6) or MNN (27.0) (Figure 2F). Besides examining the null, we also examined alternatives by performing DE analysis between two distinct cell types (alpha and beta cells) in two different ways: comparing the two cell types in the same dataset or comparing one cell type from one dataset with the other cell type from another dataset. Intuitively, if batch effects are properly removed through data integration, then the DE genes obtained in these two distinct ways would be similar to each other. Indeed, the two lists of the top 100 DE genes obtained from VIPCCA have high overlap (Jaccard index = 0.71), much more so than that obtained from the other methods (Scanorama, 0.32; scVI, 0.26; Seurat V3, 0.13; MNN, 0.01) (Supplementary Figure S3). Overall, VIPCCA facilitates the integration across multiple datasets collected from different samples and different technologies, leading to more reliable and robust downstream analyses.

### VIPCCA effectively integrates datasets with partially overlapped cell types

**Figure 3.**
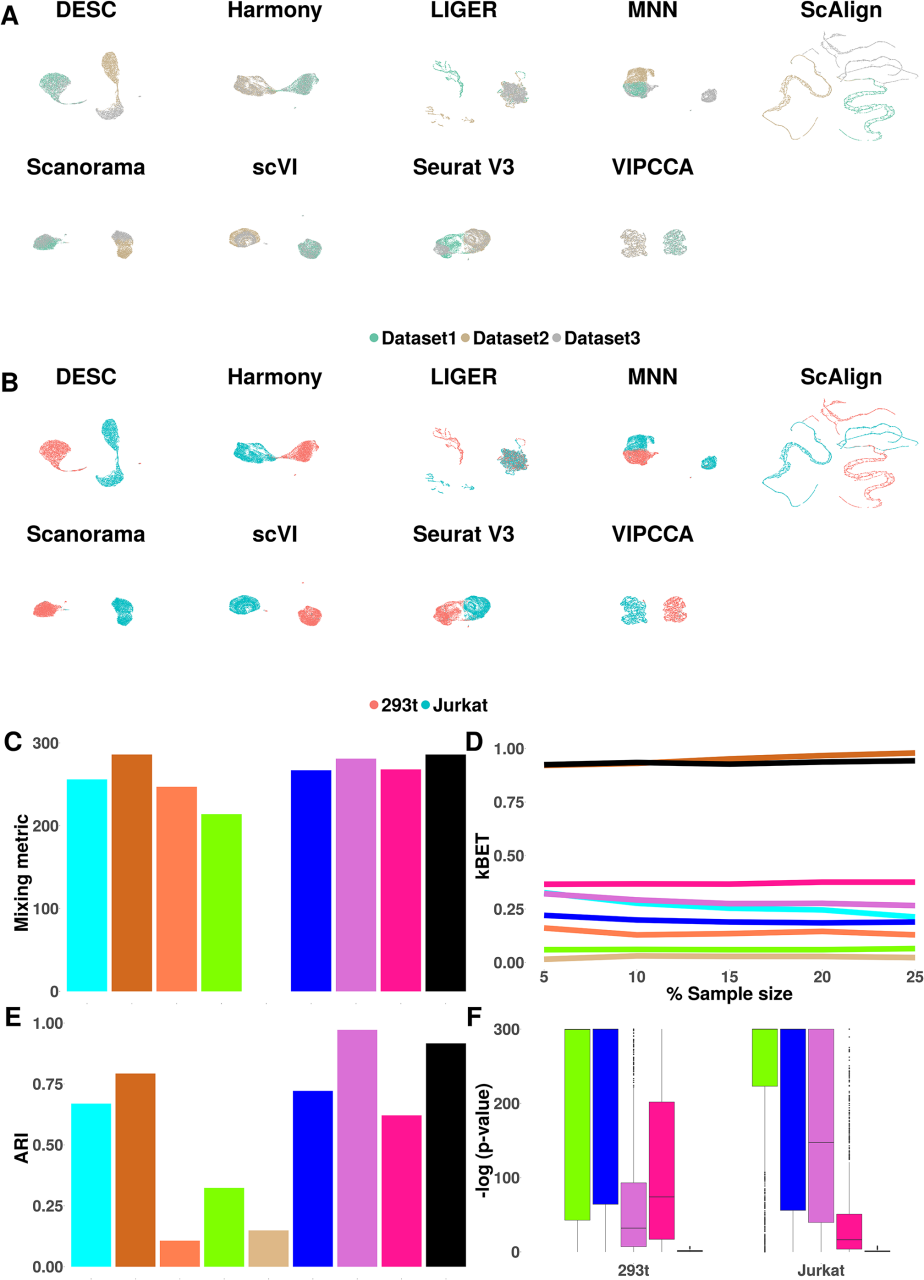
Integration of three scRNA-seq datasets with partially overlapped cell types. The examined datasets include one with 293T cells, one with Jurkat cells, and one with a 50:50 mixture of the two cell types. The examined integration methods including DESC, Harmony, LIGER, MNN, scAlign, Scanorama, scVI, Seurat V3, and VIPCCA. UMAPs are used for visualizing integration results by either batches (**A**) or cell types (**B**). Overall integration quality is measured by four metrics: mixing metric (**C**), kBET acceptance rate (**D**), adjusted rand index (ARI) (**E**) and detection of differentially expressed genes (**F**).

Our second data application examines the setting when cell types are partially overlapped across datasets, where some cell types are dataset-specific. Aligning partially overlapped datasets has been a major challenge for many single cell alignment methods. Here, we considered three published scRNA-seq datasets (31): one contains 2885 293T cells; one contains 3285 Jurkat cells; and one contains a 50:50 mixture of 1605 293T and 1783 Jurkat cells; all measured on 32 738 genes. We apply VIPCCA along with the other methods to align these three different datasets. UMAP visualization shows that both VIPCCA and Harmony successfully integrated and mixed the three datasets together, with a clear separation between the two cell types (Figure 3A, B). Seurat, scVI, DESC and Scanorama also separated cells from all three datasets into two cell types, though were unable to mix well the cells of the same cell type from different datasets. LIGER, scAlign, and MNN, on the other hand, failed to separate cells into the two cell types. Further quantification supports the visualization results. Specifically, VIPCCA and Harmony mixed cells within each cell type well, more so than the other methods: the mixing metric score is 286 for both methods, but range from 281 (scVI) to 0 (for scAlign) for the remaining seven methods (Figure 3C); VIPCCA and Harmony also achieve higher kBET acceptance rates across both cell types as compared to the remaining methods (Figure 3D, Supplementary Figure S4a). In addition, scVI and VIPCCA achieves accurate cell type clustering (ARI = 0.97, 0.92), with its performance followed by Harmony (0.79) and the other methods (range from 0.11 to 0.72) (Figure 3E); however, scVI achieves a poor mixing metric in terms of median kBET acceptance rate (0.28) across a range of sampling rate from 5% to 25%, which is much lower than that of VIPCCA (0.93).

We again evaluated the effectiveness of VIPCCA in recovering gene expression through DE analysis. We first examined the null setting by performing DE analysis on cells from the same cell type between the two corresponding datasets. Similar to what was observed in the first data application, the median −log_10_*P*-value from DE analysis on VIPCCA recovered data is low (0.9), much lower than that from Seurat (35.3), scVI (66.5), Scanorama (300) and MNN (300; Figure 3F). We also performed two additional sets of DE analyses: one between the two cell types in the mixed datasets; and the other between the two cell types from the two datasets. Consistent with the first data analysis, we found that the two lists of the top100 DE genes obtained from VIPCCA also have high overlap (Jaccard index = 0.82), more so than the other four methods (scVI, 0.79; Scanorama, 0.63; MNN, 0.53; Seurat, 0.07) (Supplementary Figure S4b). Overall, VIPCCA generates high quality integrated data for reliable and robust downstream analyses, even though partial overlaps across experiments are known to make comparison and integration particularly challenging.

### VIPCCA offers a neat solution to align scATAC-seq with scRNA-seq data

Previous methods are predominantly developed and optimized for scRNA-seq datasets, thus can be suboptimal or even inappropriate for integration across data types. In our third data application, we examined the task of integrating two different data types, a scRNA-seq data and a single cell ATAC-seq (scATAC-seq) data. Our goal is to perform reference-based cell type inference to annotate cell types in the scATAC-seq data based on the cell types in the scRNA-seq data. In particular, we examined a scRNA-seq data of 19 089 genes measured on 9432 cells and a scATAC-seq data of 89 796 peak measurements on 7866 nuclei, both collected on human peripheral blood mononuclear cells (PBMCs) (42). Standard alignment methods are not directly applicable for aligning scATAC-seq with scRNA-seq data and/or for performing subsequent cell type inference in scATAC-seq due to the distinct data structures in the two data types (17). So far, only Seurat implements cell type inference for scATAC-seq data through the alignment with scRNA-seq data. Different from the Seurat alignment algorithm used for scRNA-seq only datasets examined in previous examples, a reference-based cell type inference is introduced, which relies on a complicated procedure that weaves transfer learning, latent semantic indexing and anchor detection (17). We compared the reference-based inference algorithm in Seurat with VIPCCA for across data type integration.

**Figure 4.**
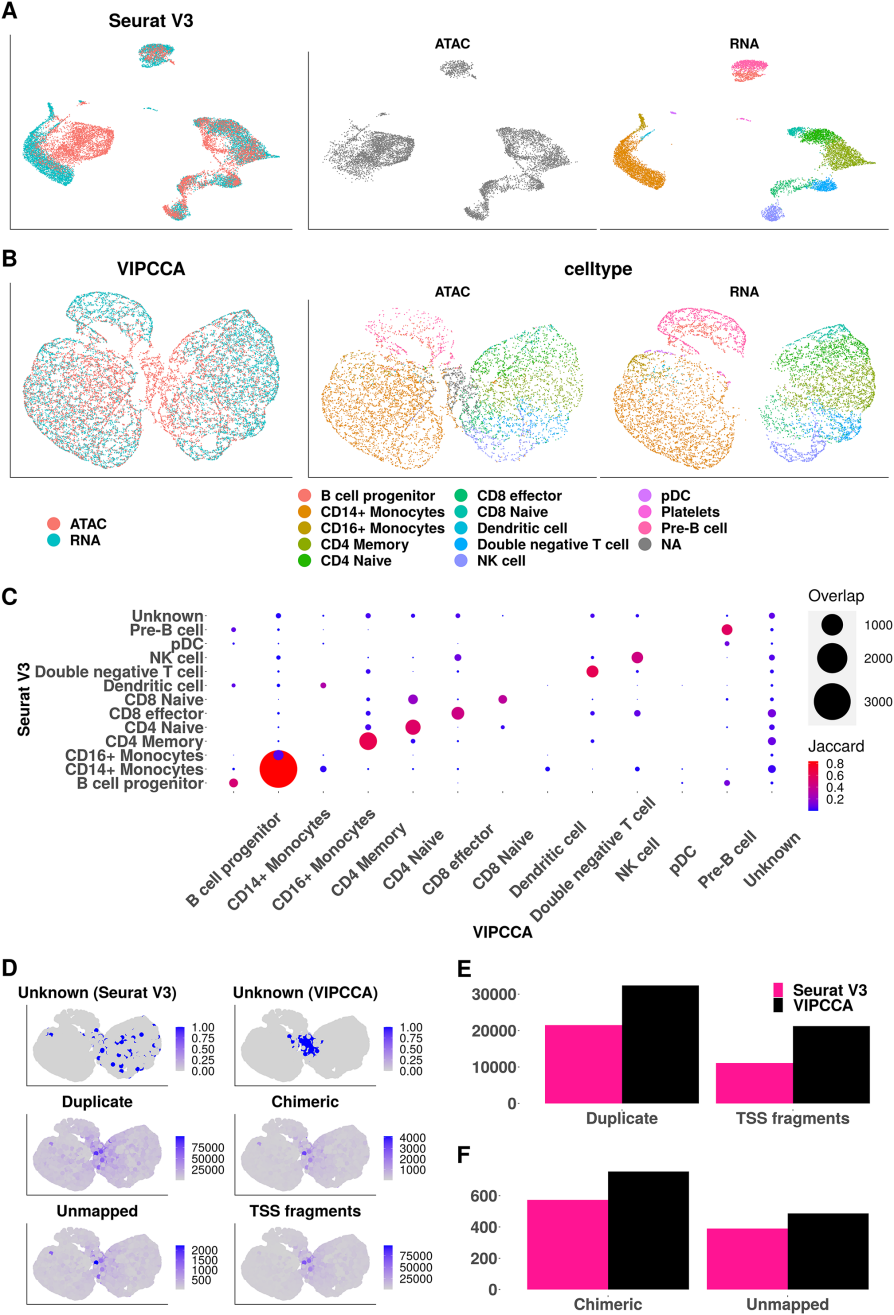
Integration of scRNA-seq and scATAC-seq datasets on PBMCs. (**A**) and (**B**) show UMAP visualizations of scRNA-seq and scATAC-seq data based on the cell embeddings obtained from Seurat and VIPCCA, respectively. Each dot represents a cell/nucleus colored by either datasets (left) or cell types (middle and right). We are unable to color cell types predicted by Seurat V3 because it relies on a completely different strategy to infer cell types that do not look well on the low dimensional space. (**C**) compares results between Seurat V3 and VIPCCA by visualizing the number of overlapped cells and the Jaccard index for each pair of predicted cell types. (**D**) shows the distribution of cells on the UMAP space that are enriched with unknown nuclei by VIPCCA, unknown nuclei by Seurat V3, duplicate mapped read-pairs, chimerically mapped read-pairs, read-pairs with at least one end not mapped, and fragments overlapping with TSS regions. (**E**) shows the mean duplicate mapped read-pairs and mean TSS fragments in the unassigned cells by the two methods. (**F**) shows the mean chimerically mapped read-pairs and unmapped read-pairs in the unassigned cells by the two methods.

In the analysis, we found that VIPCCA can be directly used to align these two data types without employing any complicated ad-hoc algorithmic procedure as used in Seurat. Indeed, UMAP visualization shows that VIPCCA mixes two data types well, more so than that obtained by Seurat (Figure 4A,B). Both mixing metric score (VIPCCA: 287.5; Seurat: 260.5) and kBET acceptance rate (Supplementary Figure S5a) confirms such observation. VIPCCA assigned 7233 out of the 7866 nuclei (92.0%) into a known scRNA-seq cell type and assigned the remaining 633 nuclei as unknown. The known cell types in scATAC-seq inferred by VIPCCA display expected chromatin accessibility pattern that are consistent with bulk ATAC-seq data (42) (Supplementary Figure S5b) and largely consistent with the cell types inferred by Seurat (Figure 4C). A careful examination of the chromatin accessibility profile near eight known cell type marker genes (17,40) revealed similarity between VIPCCA and Seurat based inferences (Supplementary Figures S6−S9), along with subtle but important differences: for example, a clear ATAC-seq peak is observed inside the pDC marker gene LYZ (41) in pDC cells inferred by VIPCCA (Supplementary Figure S7a), but not observed in pDC cells inferred by Seurat (Supplementary Figure S7b). On the other hand, the unknown scATAC-seq cell types inferred by VIPCCA are notably different from that inferred by Seurat (*n* = 274): the unassigned cells by VIPCCA are typically of low data quality as they are characterized by an excessive number of duplicate mapped read-pairs, chimerically mapped read-pairs, read-pairs with at least one end not mapped, or fragments overlapping with TSS regions, much more so than the unassigned cells by Seurat (Figure 4D−F). Overall, the above analyses strongly support the effectiveness of VIPCCA in integrating scATAC-seq data with scRNA-seq data.

### VIPCCA enables scalable integration of millions of cells

**Figure 5.**
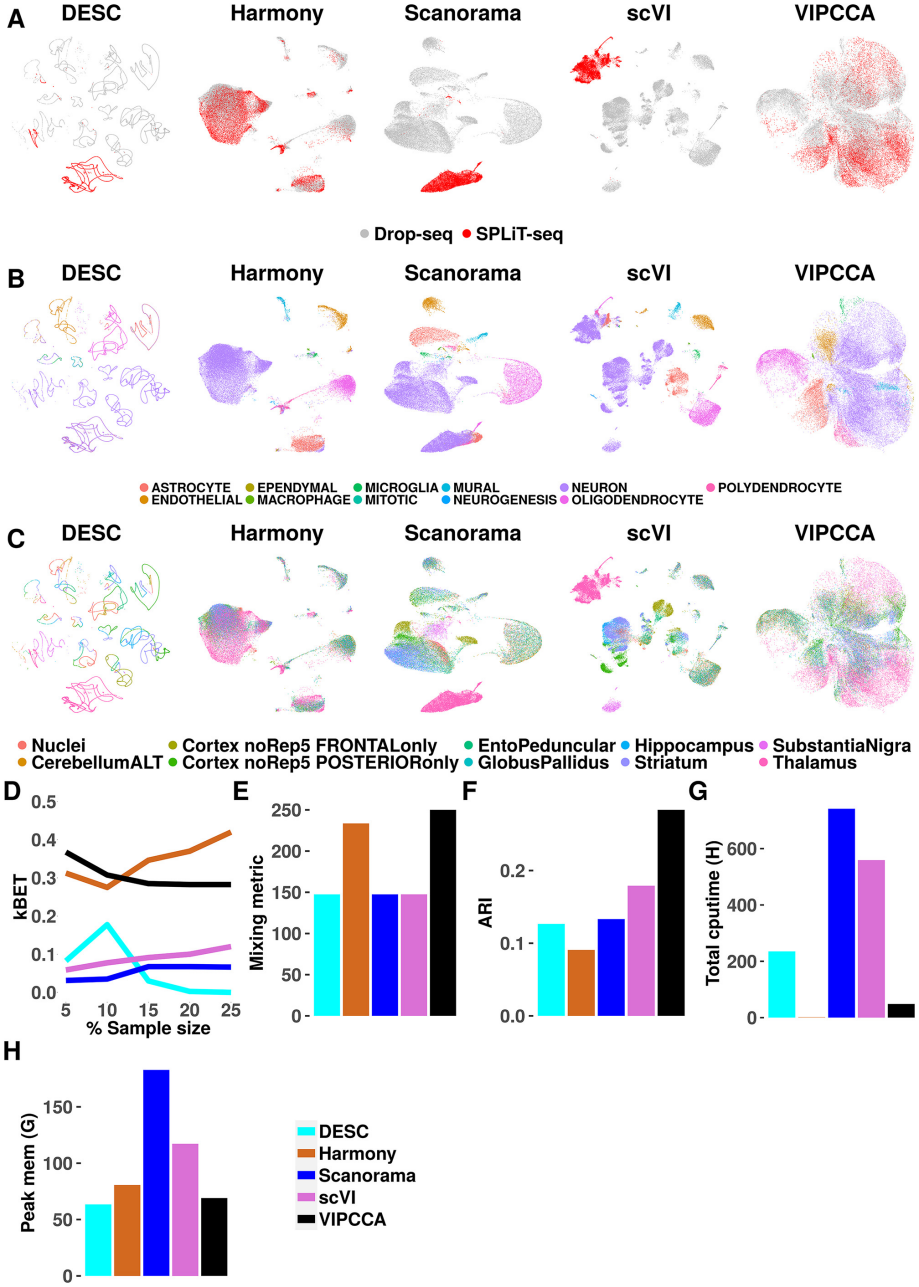
Integration of over a million cells from Drop-seq and SPLiT-seq from mouse brain and spinal cord. Examined integration methods include DESC, Harmony, scVI, Scanorama, and VIPCCA. Each of the 9 CNS regions and the nuclei were downsampled to 10% for UMAP visualization and visualize results are shown by technologies (**A**), cell types (**B**) or regions (**C**). Cells from each dataset are colored by several major cell types according to the cell type annotations from the original publications. Overall integration quality is measured by three metrics: the average kBET acceptance rate (**D**), mixing metric (**E**) and ARI (**F**). Total cpu time (**G**) and peak memory usage (**H**) are also recorded for different methods, all measured on a workstation with 24 cores of Intel(R) Xeon(R) CPU E5-2620 v2, with 256G memory, and with one GPU of GeForce GTX 2080 Ti.

Scalability is a key bottleneck for single cell alignment methods. In the fourth data application, we exploited with a large-scale integrative analysis of 1 099 538 cells from two studies: a scRNA-seq study on nine regions in the mouse brain with Drop-seq (32) and a single nuclei RNA-seq study on the mouse brain and spinal cord with SPLiT-seq (33). Only five methods (DESC, Harmony, Scanorama, scVI and VIPCCA) are applicable for such large-scale integrative analysis. In the analysis, UMAP visualization shows that both Harmony and VIPCCA mixed cells from the two datasets well and were able to correctly segregate cells by both cell types and regions, more so than DESC, Scanorama and scVI (Figure 5A−C). In contrast, DESC produces multiple curvature patterns that appear to be highly artificial while Scanorama segregates cells into their original regions but not cell types. Quantification supports the same conclusion in terms of mixing: both VIPCCA and Harmony lead to higher kBET acceptance rates than DESC, Scanorama and scVI (Figure 5D); and VIPCCA achieves the highest mixing metric score (250.0), which is followed by Harmony (233.5), Scanorama (147.5), scVI (147.5) and DESC (147.5) (Figure 5E). VIPCCA also infers cell types reasonably accurately (ARI = 0.28), more so than scVI (0.18), Scanorama (0.13), DESC (0.13), and Harmony (0.09) (Figure 5F). The cell types inferred by VIPCCA are enriched with cell type specific markers as one would expect (Supplementary Figures S10 and S11). In addition, both VIPCCA and Harmony are more computationally efficient than the other three (Figure 5G, H). Overall, the results suggest that VIPCCA is both effective and computationally efficient for large-scale data integration.

### VIPCCA integration facilitates trajectory inference

**Figure 6.**
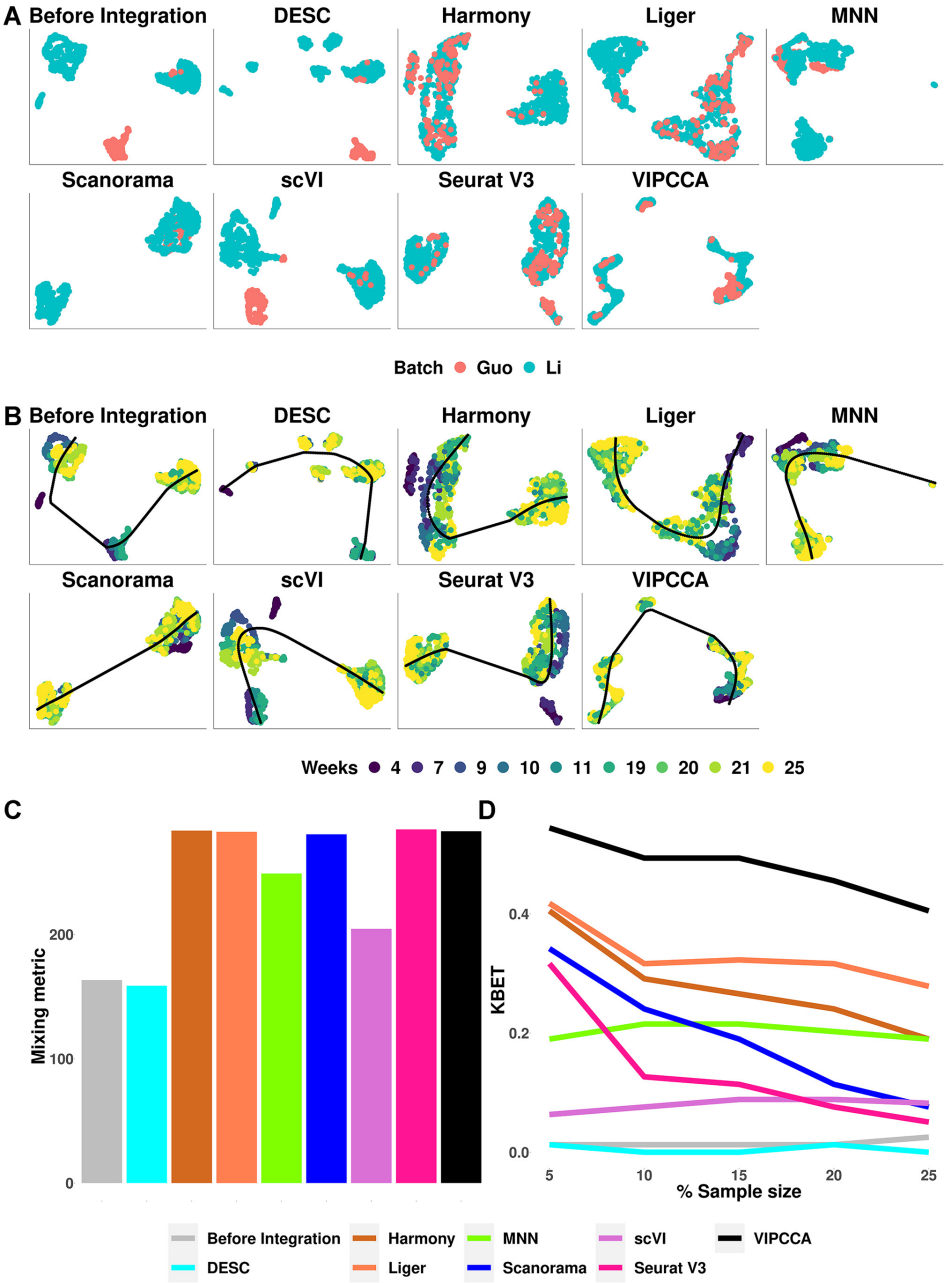
Integration of two datasets of human male germline cells that were collected on a series of time points from 4 weeks to 25 weeks. The examined integration methods include DESC, Harmony, LIGER, MNN, Scanorama, scVI, Seurat V3 and VIPCCA. Data are visualized by either batches (**A**) or collection time (**B**) on the UMAP space before integration and after integration using different integration methods. Slingshot was applied to perform trajectory inference based on the cell embeddings in reduced dimensional space inferred by each alignment method. Integration quality is further evaluated by the mixing metric (**C**) and kBET acceptance rate (**D**).

Trajectory inference refers to the process of determining the pattern of a dynamic process experienced by cells and subsequently ordering cells based on their progression. Samples used for trajectory inference are often expected to contain cells with distinct biological states or under different developmental stages, the alignment of which can be particularly challenging. In the last data application, we examined the performance of VIPCCA to aligning samples that contain cells with heterogeneous and dynamic states. Specifically, we examined two-time series scRNA-seq studies on human primordial germline cells (PGCs): one with 666 female cells (5–26 weeks) and 649 male cells (4–25 weeks) measured on 24 153 genes (35), another with 83 female cells (4–17 weeks) and 141 male cells (4–19 weeks) measured on 23 394 genes (36). PGCs are the embryonic precursors of the gamete, with male and female PGCs undergoing several distinct sequential phases during development (35,36). Because of the distinct transcriptomic features of female and male PGCs after 4 weeks of gestation (35), we separately integrated the female and male PGCs from the two collected datasets. UMAP visualization shows that five methods (Harmony, LIGER, Scanorama, Seurat V3, VIPCCA) can mix the two datasets well (Figure 6A, Supplementary Figure S12a). We applied trajectory inference on the integrated data using Slingshot (39). The alignment results suggest that VIPCCA can successfully identify a linear lineage on both the male and female data (Figure 6B, Supplementary Figure S12b). Our results also demonstrated that the early-stage cells span from 4W to 25W in male PGCs, and from 4W to 26W in female PGCs (Figure 6B, Supplementary Figure S12b), which is consistent with the results reported in (35). The mixing metric score for the five methods in male PGCs are in the range of 280–285, which are higher than that obtained before integration (163.3), by DESC (158.8), by scVI (204.5) or by MNN (249.0) (Figure 6C). The mixing metric score for the five methods in female PGCs are in the range of 271–282, which are again higher than that obtained before Integration (200.0), by DESC (225.0), by scVI (235.0) or by MNN (246.0) (Supplementary Figure S12c). VIPCCA also achieves the highest kBET acceptance score in both male and female cells, with its performance followed by LIGER, Harmony, and the other methods (Figure 6D, Supplementary Figure S12d). Careful examination of known early vs late stage PGC marker genes confirms the expected segregation of late-stage PGCs from early-stage PGCs in VIPCCA aligned data (Supplementary Figures S13−S14). Early-stage male PGCs (mitotic stage) clearly expressed both early markers (36) (e.g. *KIT*) and pluripotency markers (e.g. *POU5F1*, *NANOG*, *KLF4*) (Supplementary Figure S13) and displayed substantial transcriptomic heterogeneity. For example, the two sub-clusters of early-stage male PGCs are clearly visible and correspond to migrating PGCs and gonadal mitotic (Figure 6B). Late-stage male PGCs (mitotic arrest stage) expressed well characterized marker genes in male mitotic arrest phase (e.g. *NANOS2* and *CDK6*) (Supplementary Figure S13). Therefore, VIPCCA integration reveals true biological states during cell development and facilitates trajectory inference.

## DISCUSSION

The explosion of single cell technologies brings opportunities and challenges that call for more powerful and scalable integration methods. We have presented a new method, VIPCCA, which is versatile, flexible, and broadly applicable to a wide range of single-cell data integration tasks. Compared with previous methods, VIPCCA’s contributions are 3-fold: (i) VIPCCA builds upon a data generative model, which yields normalized results leading to better interpretation and more reliable downstream analyses, without any post hoc data processing; (ii) non-linearity modeling, inspired by the latest practice from deep learning community, enables VIPCCA to capture complex data structure, which has been shown highly adaptive across platforms, data types and cell states; (iii) fueled by variational inference computational techniques, VIPCCA implements computationally efficient algorithms that are scalable to millions of cells, placing the incoming large-scale atlas datasets within reach.

We expect VIPCCA to be broadly used for various integrative analyses such as reference atlas assembly and transfer annotations of reference single-cell data across experiments and modalities. Although we have only demonstrated the ability of VIPCCA in integrating data types through the analysis of scRNA-seq and scATAC-seq data, our integration strategy has the potential for integrating other data types such as the recent spatially resolved transcriptomics datasets with single cell RNAseq data (45,46). Specifically, for single-cell resolution spatial transcriptomics such as MERFISH, SeqFISH, SeqFISH + and STARmap, we can directly treat each location of the spatial transcriptomics data as a single cell and proceed with VIPCCA alignment as if the spatial transcriptomics data is a single cell RNA-seq data. For regional resolution spatial transcriptomics such as 10x visium, Slide-seq and high-definition spatial transcriptomics (HDST), each spatial location would contain potentially a few to a few dozen single cells. For these regional resolution data, we can attempt to represent each location of the spatial transcriptomics data by the major cell type residing there and proceed with VIPCCA alignment by treating the spatial transcriptomics data approximately as a single cell data. Alternatively, we can extend VIPCCA by introducing a one-to-many mapping matrix, which can be used to relate each spatial location of the spatial transcriptomics to multiple cells in the scRNA-seq data to facilitate data alignment. With similar approaches, VIPCCA can be extended to integrate two or more spatially resolved transcriptomics datasets that are collected on adjacent or similar tissue sections. These applications could potentially offer us the promise of understanding cellular and spatial heterogeneity of a complex tissue or a disease tissue in the spatial context. Further, the continued development of complementary tools will facilitate comparative analysis of two or more samples under different biological conditions (e.g. tumor samples and tumor samples with drug treatment), which would benefit us with insights for understanding how cells respond to therapy. VIPCCA allows us to take advantage of the rapid pace of single-cell technological development and the vast accumulation of single cell data types for gaining unique insights into the cellular and spatial heterogeneity of complex tissues.

Like some other existing single cell alignment methods (19,47,48), we have primarily focused on using a Gaussian noise distribution to model normalized gene expression or gene activity data that are converted from the original count data. Modeling normalized data is computationally much more tractable than modeling the original count data using over-dispersed or zero inflated Poisson models (e.g. negative binomial, Poisson mixed models, zero-inflated negative binomial etc.) (49–51). However, scRNA-seq and scATAC-seq data are of count nature. Because of the relatively low sequencing depth of single cell sequencing, accounting for the mean and variance relationship by modeling the original count data directly often has added benefits (52). Therefore, extending our method to align the original count data from scRNA-seq and scATAC-seq directly while properly accounting for the over-dispersion or dropout events will likely improve alignment accuracy further.

## DATA AVAILABILITY

The software implementing VIPCCA, all source code and data sets used in our experiments have all been deposited at https://github.com/jhu99/vipcca and https://github.com/jhu99/vipcca_paper.

## Supplementary Material

gkab1147_Supplemental_Files
