# Supplementary material for: Effective and scalable single-cell data alignment with non-linear canonical correlation analysis
Source: Nucleic Acids Res. 2021 Dec 6;50(4):e21. doi: 10.1093/nar/gkab1147 (PMC8887421; doi:10.1093/nar/gkab1147)
Supplement: gkab1147_Supplemental_Files [file gkab1147_supplemental_files.zip › Supplementary Figures-20210928.docx]

# Supplementary Figures


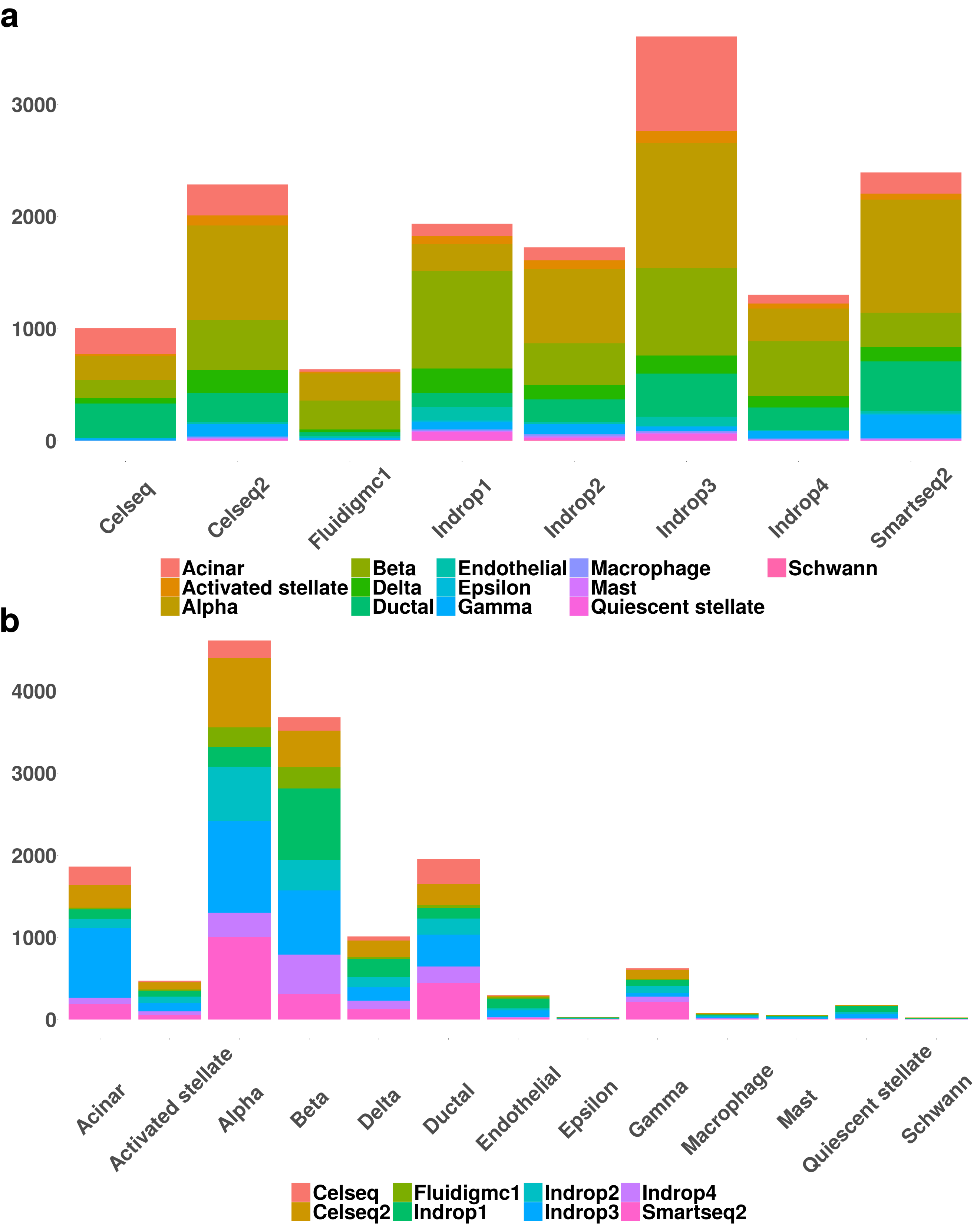


Figure S1. Cell type composition in each batch (a) and batch composition in each cell type (b) in the first data application. The cell type and batch labels are obtained from the SeuratData R package (“panc8”).


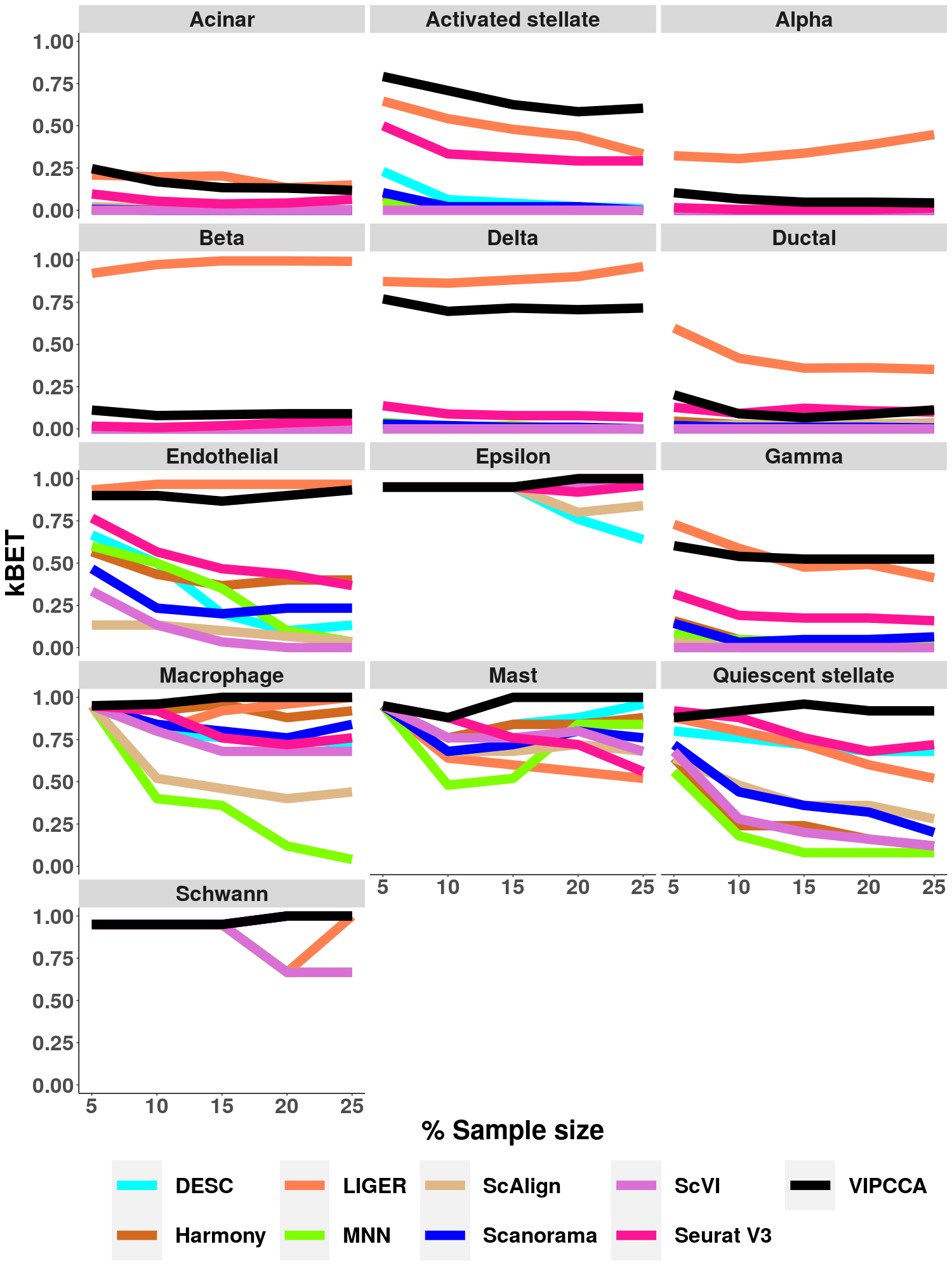


Figure S2. kBET acceptance rate for each of 13 cell types in the integrated pancreatic datasets is displayed across a range of neighborhood size equaling to 5% to 25% of the sample size. Each line represents the mean acceptance rate calculated based on 100 repeated kBET runs. Each color represents an integration method.


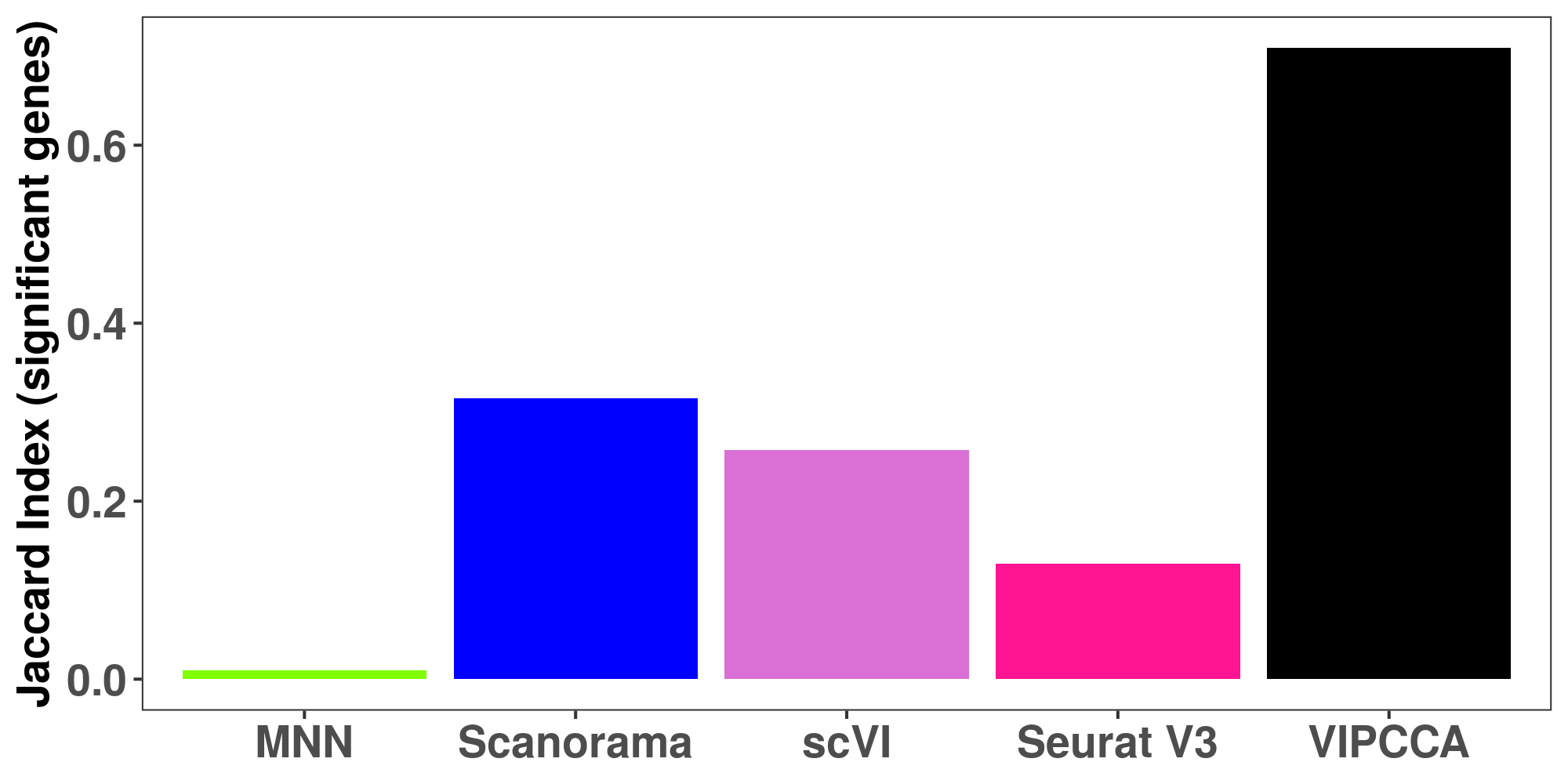


Figure S3. Jaccard index between two sets of the top 100 differentially expressed genes detected by different alignment methods in the first data application. The DE analysis is performed between two cell types (alpha and beta cells) in two different ways: alpha vs beta cells within the celseq dataset (Scenario 2); alpha from celseq vs beta from celseq2 (Scenario 3). The two sets of differentially expressed genes are expected to be similar to each other if batch effects were effectively removed.

*
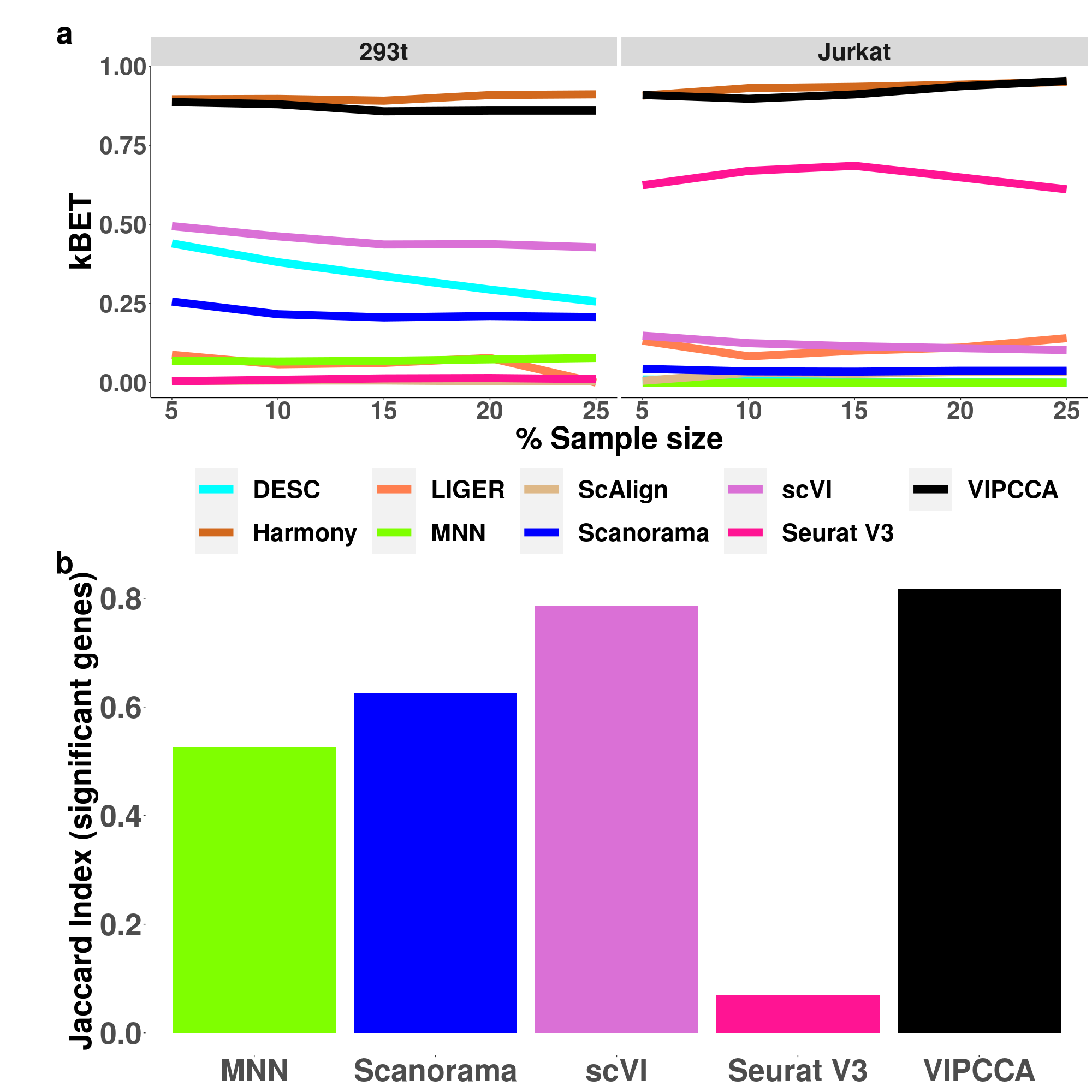
*

Figure S4. (a) kBET acceptance rate for the 293t and Jurkat cells from the three datasets in the second data application is shown across a range of neighborhood size equaling to 5% to 25% of the sample size. Each line represents the mean acceptance rate calculated based on 100 repeated kBET runs. Each color represents an integration method. (b) Jaccard index between two sets of the top 100 differentially expressed genes in Scenario 2 and Scenario 3 in the second application. We performed DE analysis between two cell types (293t and Jurkat cells) in two different ways: 293t and Jurkat cells within the mixed dataset (Scenario 2); 293t from the 293t dataset, Jurkat from the mixed dataset (Scenario 3). The two set of differentially expressed genes tend to be identical if batch effects were effectively removed.


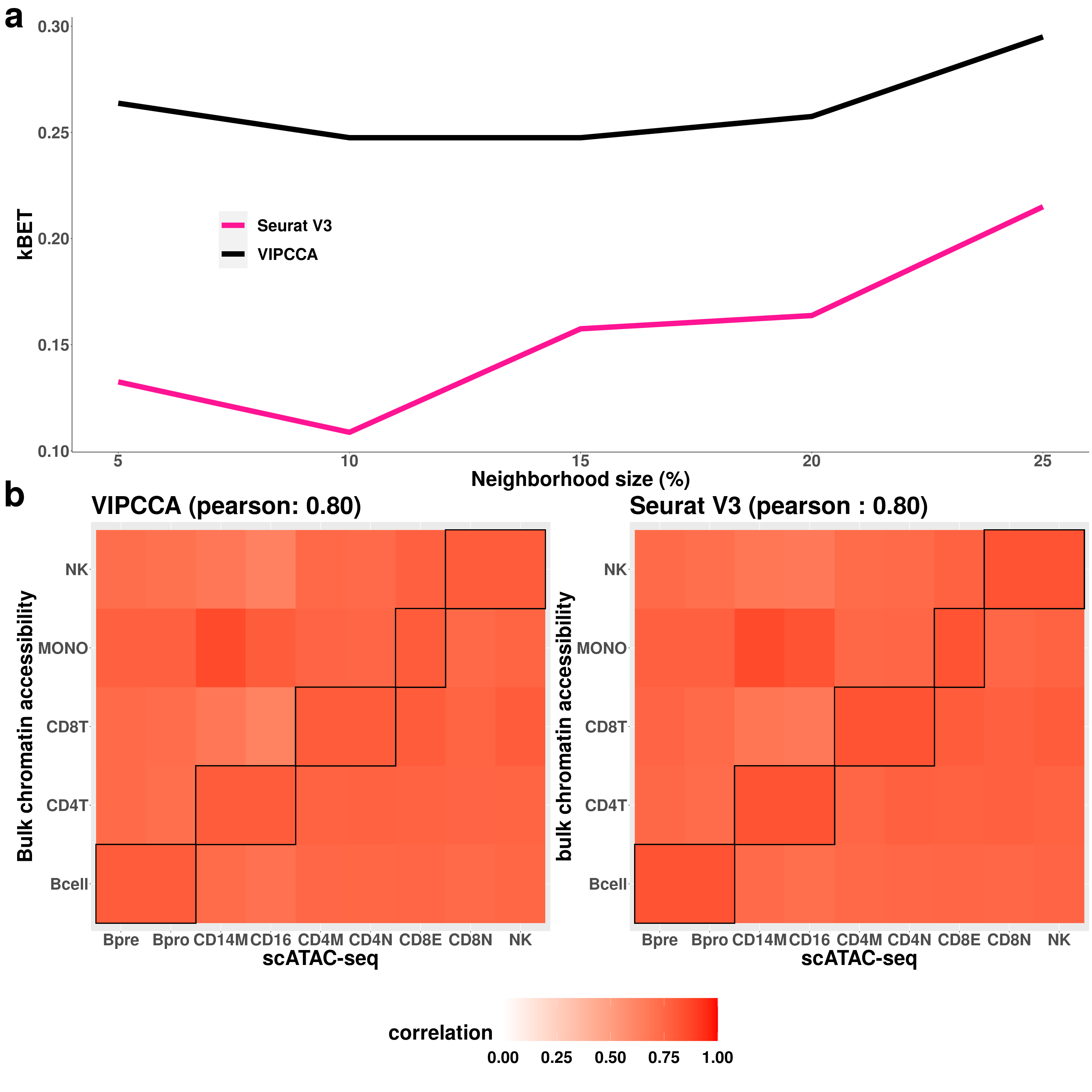


Figure S5. (a) kBET acceptance rate for cells from the integrated scRAN-seq and scATAC-seq datasets in the third data application is shown across a range of neighborhood size equaling to 5 to 25% of the sample size. Each line represents the mean acceptance rate calculated based on 100 repeated kBET runs. Each color represents an integration method. (b) Pearson correlation of the global pattern of chromatin accessibility between pseudo ATAC-seq profile and bulk ATAC-seq profile. The pseudo ATAC-seq profiles were created by pooling cells predicted by VIPCCA (b, left) and Seurat V3 (b, right).

*
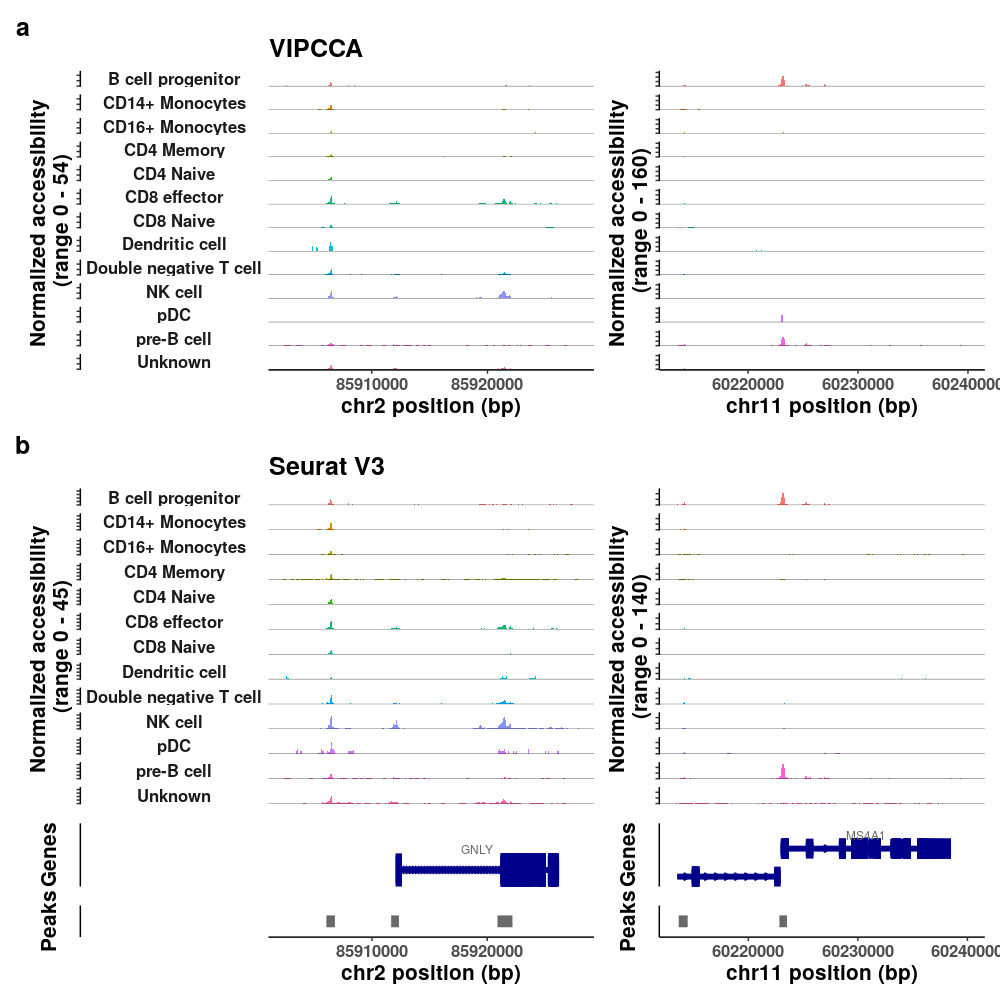
*

Figure S6. Chromatin accessibility tracks on two marker genes and its nearby flanking regions for different cell types predicted by VIPCCA (a) and Seurat V3 (b). The two marker genes include GNLY, MS4A1 (columns).


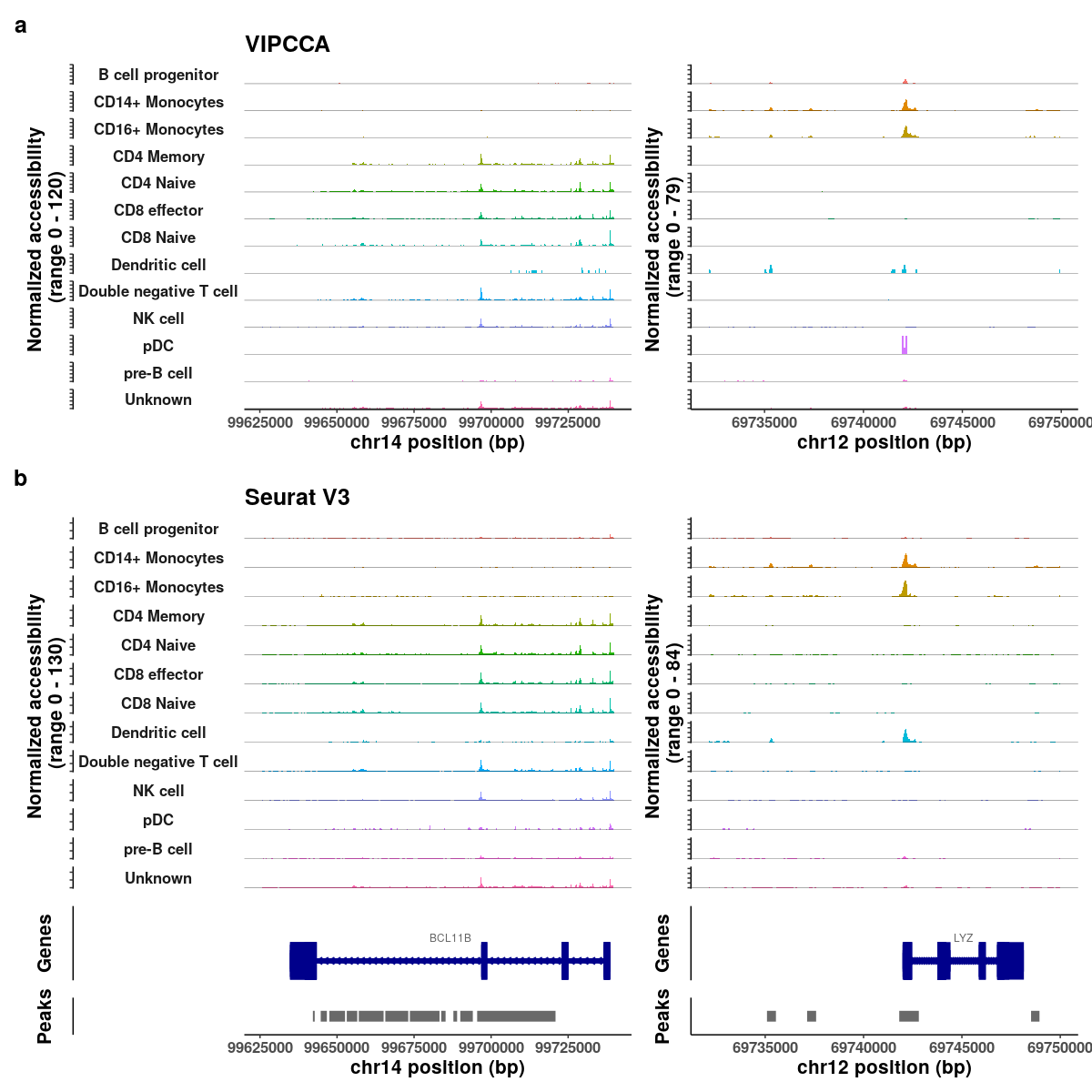


Figure S7. Chromatin accessibility tracks on two marker genes and its nearby flanking regions for different cell types predicted by VIPCCA (a) and Seurat V3 (b). The two marker genes include BCL11B and LYZ (columns).


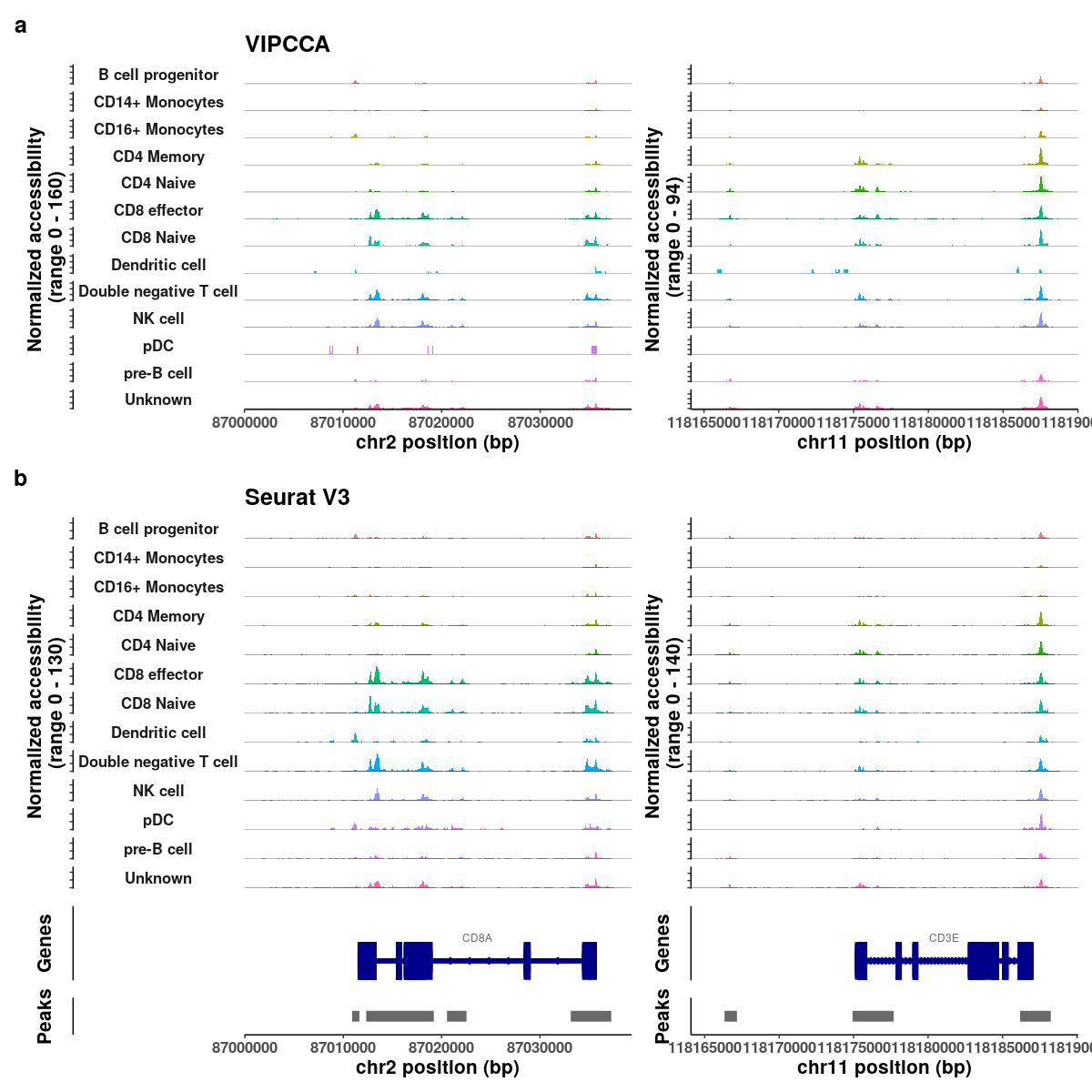


Figure S8. Chromatin accessibility tracks on two marker genes and its nearby flanking regions for different cell types predicted by VIPCCA (a) and Seurat V3 (b). The two marker genes include CD8A, CD3E (columns).


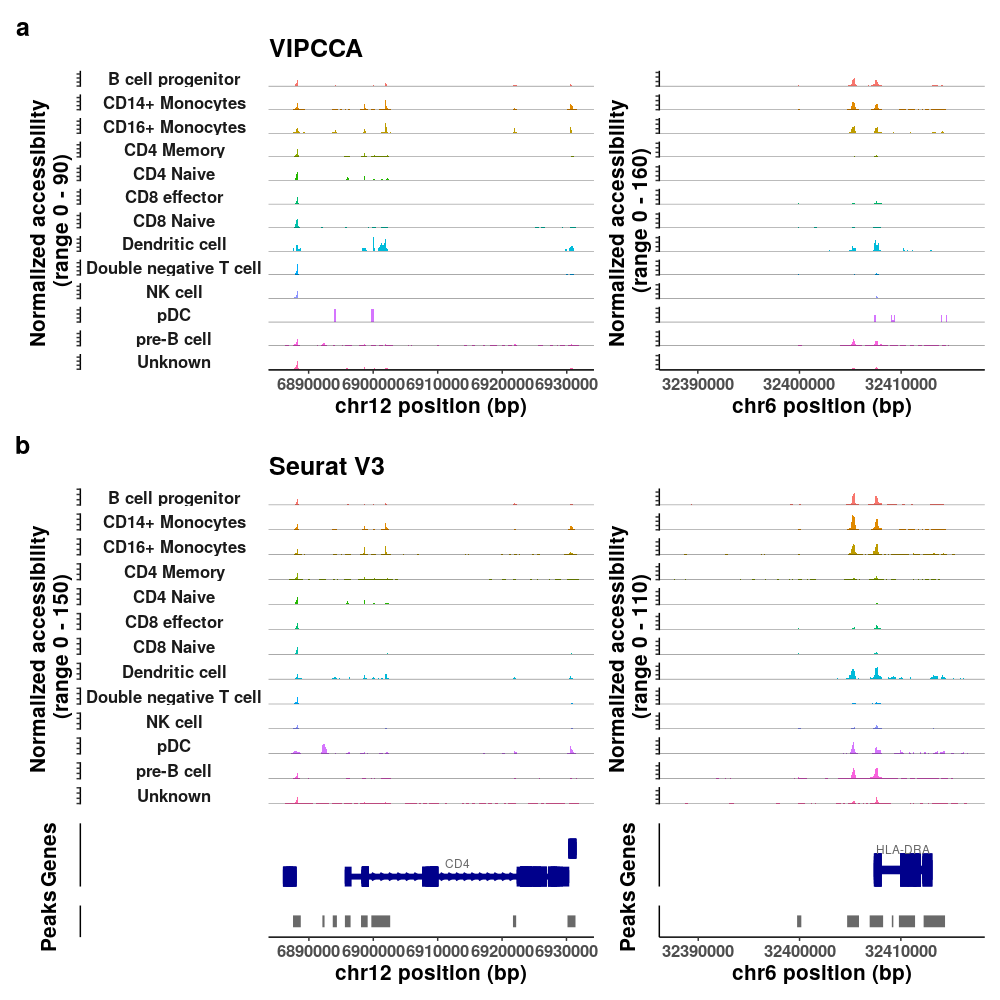


Figure S9. Chromatin accessibility tracks on two marker genes and its nearby flanking regions for different cell types predicted by VIPCCA (a) and Seurat V3 (b). The two marker genes include CD4, HLA-DRA (columns).


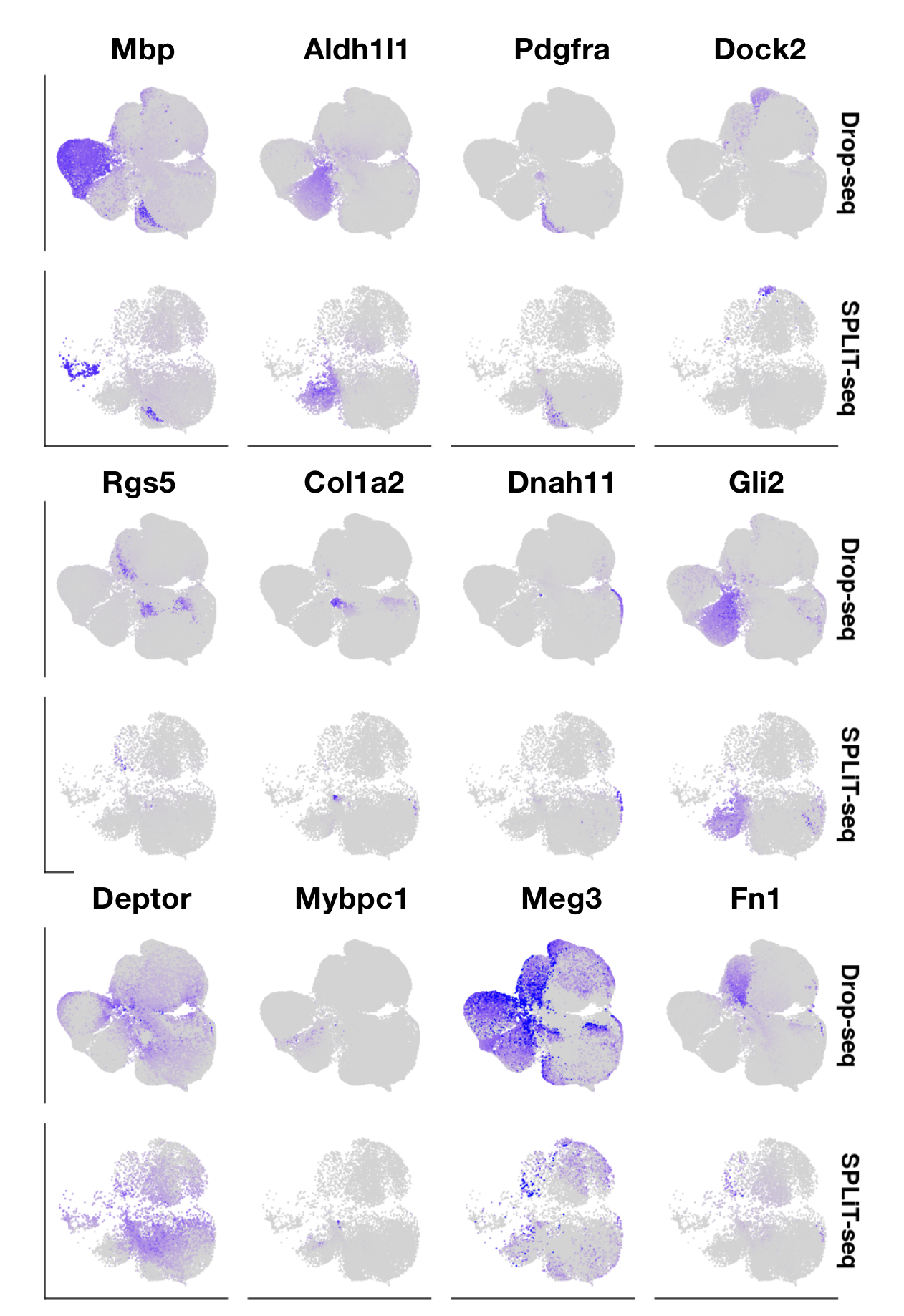


Figure S10. UMAP feature plots on 12 known marker genes. The marker genes include Mbp (oligodendrocytes), Aldh1l1(astrocytes), Pdgfra (oligodendrocyte precursor cells), Dock2 (microglia and macrophages), Rgs5 (endothelial cells and smooth muscle cells), Col1a2 (vascular and leptomeningeal cell), Dnah11 (ependymal cells), Gli2 (cerebellar granule cell in the external granule layer), Deptor (Olfactory bulb), Mybpc1 (olfactory ensheathing cells), Meg3 (neural cells), and Fn1 (hippocampus). UMAP is obtained based on aligned data from VIPCCA.


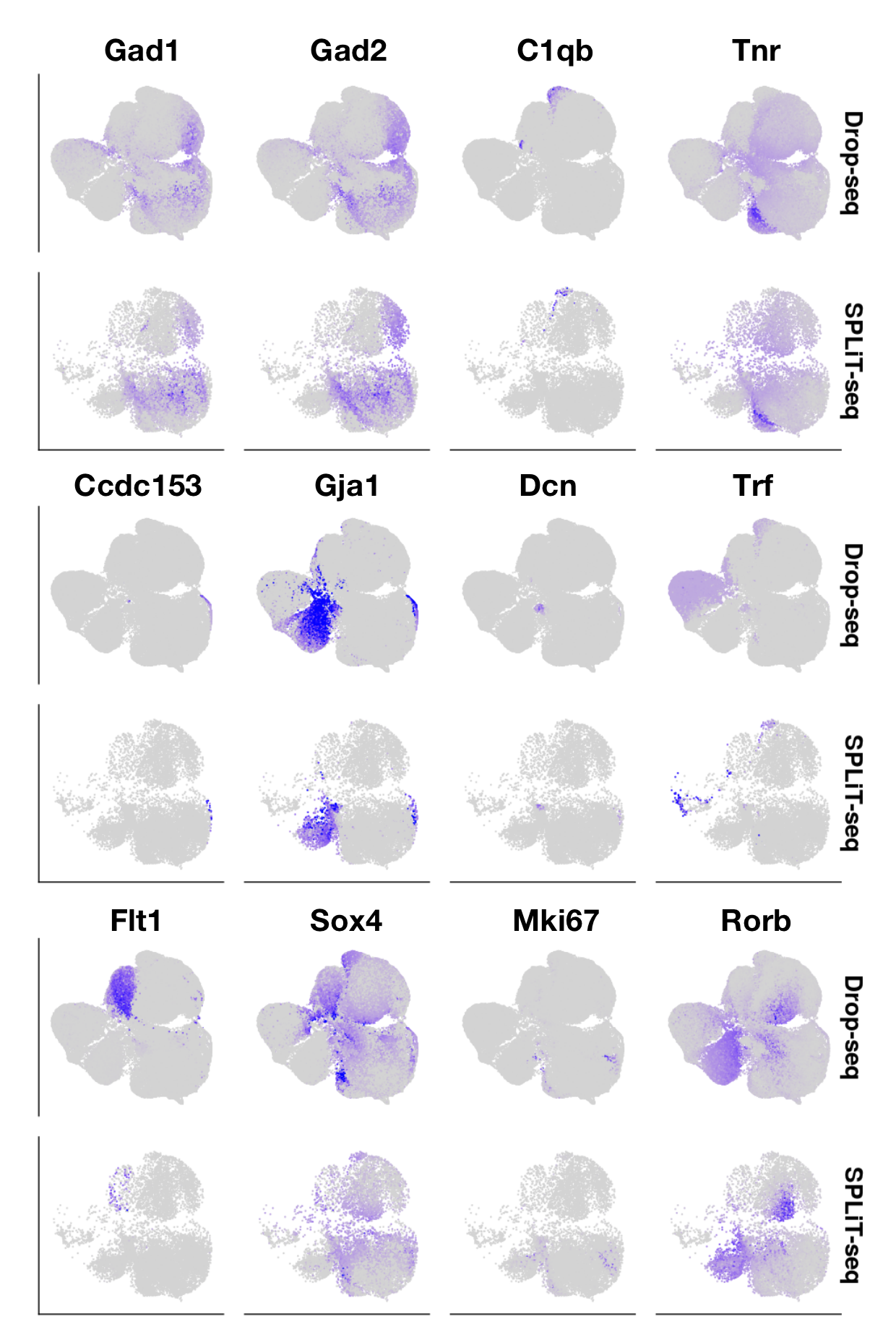


Figure S11. UMAP feature plots on 12 known marker genes. Marker genes include Gad1 and Gad2 (GABAergic neurons), C1qb (microglia and macrophages), Tnr (Polydendrocyte), Ccdc153 (Ependymal), Gja1 (astrocytes), Dcn (Endothelial_Tip), Trf (oligodendrocytes), Flt1 (Endothelial_stalk), Sox4 (Neurogenesis), Mki67 (Mitotic), Rorb (Cortical pyramidal neuronal cells in PyrL4). UMAP is obtained based on aligned data from VIPCCA.


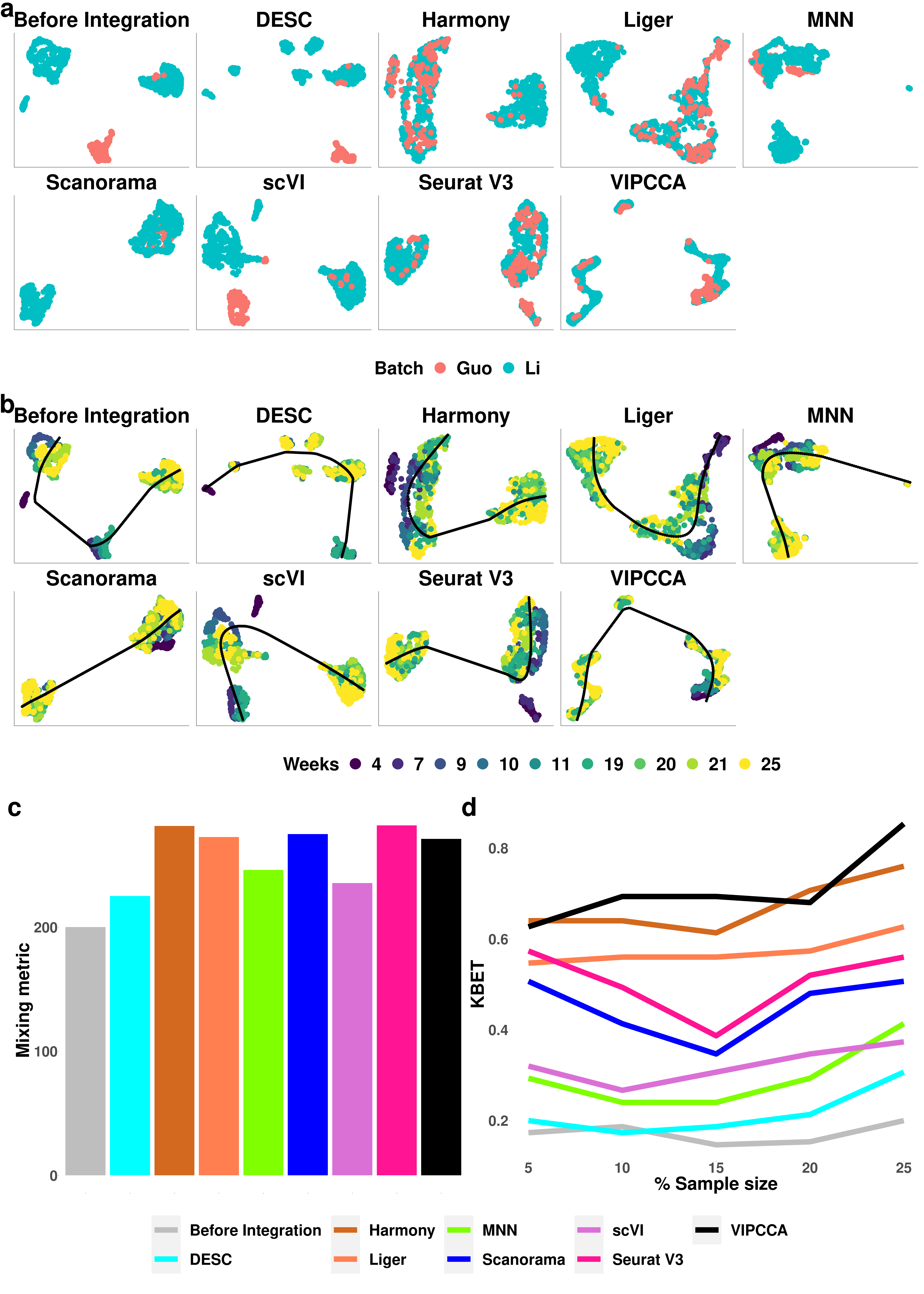


Figure S12. Integration of two datasets on human female germline cells. The human female germline cells were collected on a series of time points from 4 weeks to 26 weeks. Each cell was represented by a dot and colored by batches (a) or collection time (b) in UMAP visualization. Data without integration (i.e. Before Integration, reduced by PCA) and eight other integration algorithms were compared for performance evaluation, which include DESC, Harmony, LIGER, MNN, Scanorama, scVI, Seurat V3 and VIPCCA. Trajectory and pseudotime were inferenced by using Slingshot based on cell embeddings in reduced dimensional space of each integration algorithms. Plots in (c) and line plots in (d) show the mixing metric and kBET acceptance rate of each integrated data over a range of neighborhood size from 5 to 25% of the sample size.


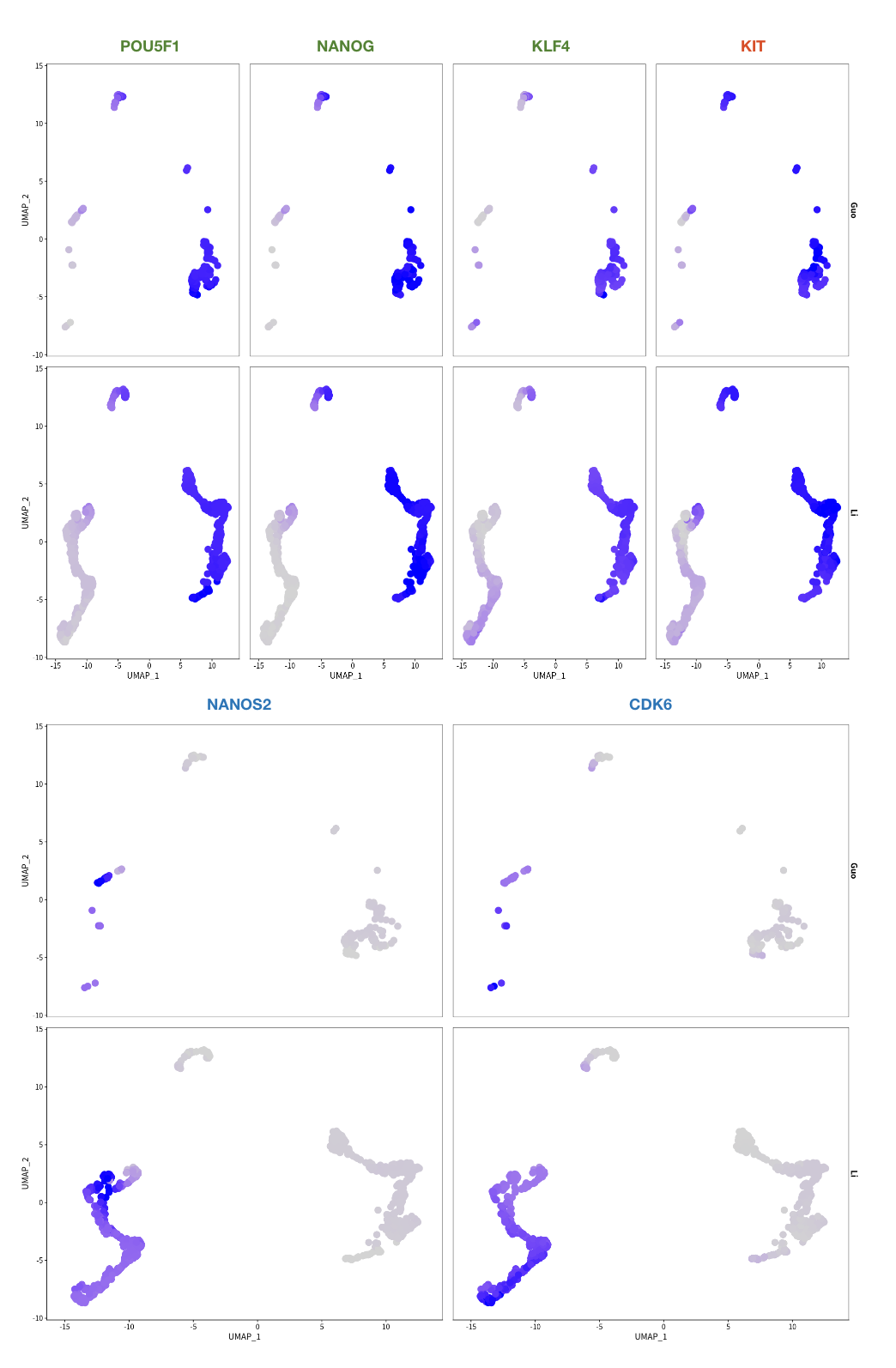


Figure S13. UMAP feature plots show six gene markers for male germline cells. The gene markers include three pluripotency markers (green；POU5F1, NANOG, KLF4), one early stage marker (red; KIT), and two late stage markers (blue; NANOS2, CDK6). UMAP is obtained based on aligned data from VIPCCA.


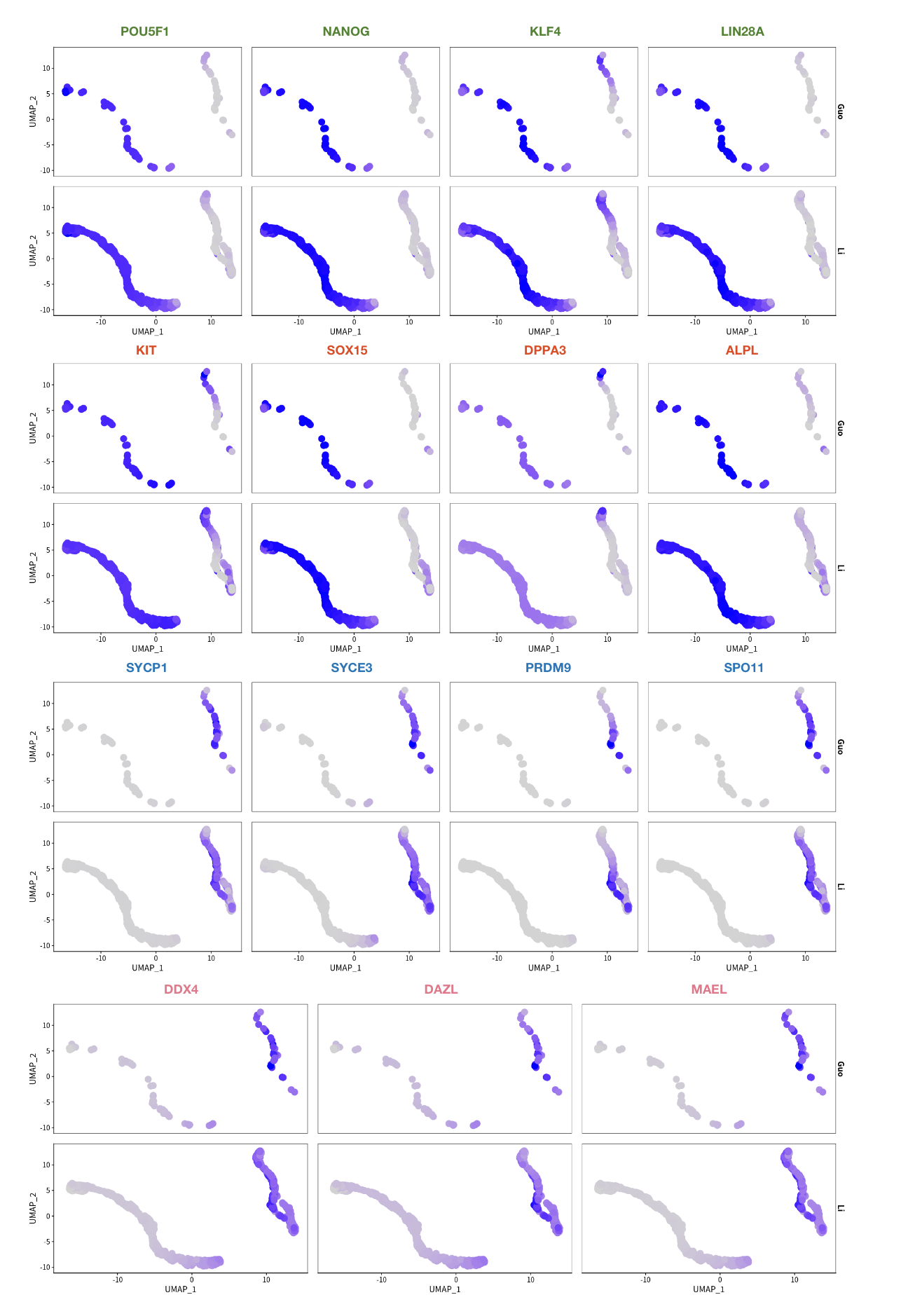


Figure S14. UMAP feature plots of fifteen gene markers for female germline cells. The gene markers include four pluripotency markers (green; POU5F1, NANOG, KLF4, LIN28A), four early stage markers (red; KIT, SOX15, DPPA3, ALPL), four meiosis markers (blue; SYCP1, SYCE3, PRDM9, SPO11), and three late stage markers (pink; DDX4, DAZL, MAEL). UMAP is obtained based on aligned data from VIPCCA.

*
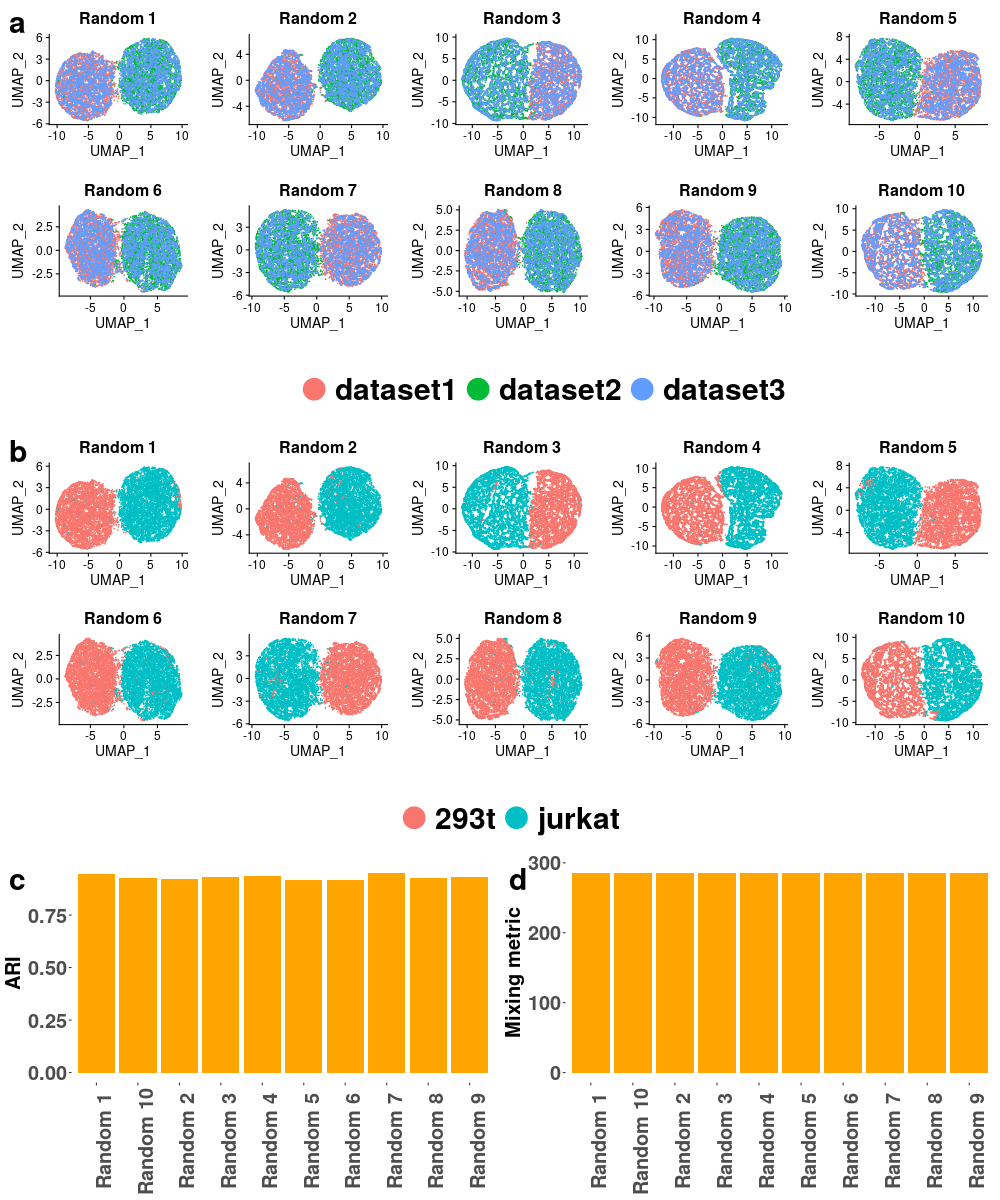
*

Figure S15 (a) UMAP visualization for the 293t and Jurkat cells from the three datasets in the second data application is shown across a range of randomly generated _­_$b^{(m)}$_._ Each dot represents a cell, colored by datasets (a) and by cell types (b). The stability of our method over the randomness of $b^{(m)}$ is measured by adjusted rand index (ARI) (c) and the mixing metric (d). ARI, mean=0.930, sd=0.0110; mixing metric, mean=286, sd=0.


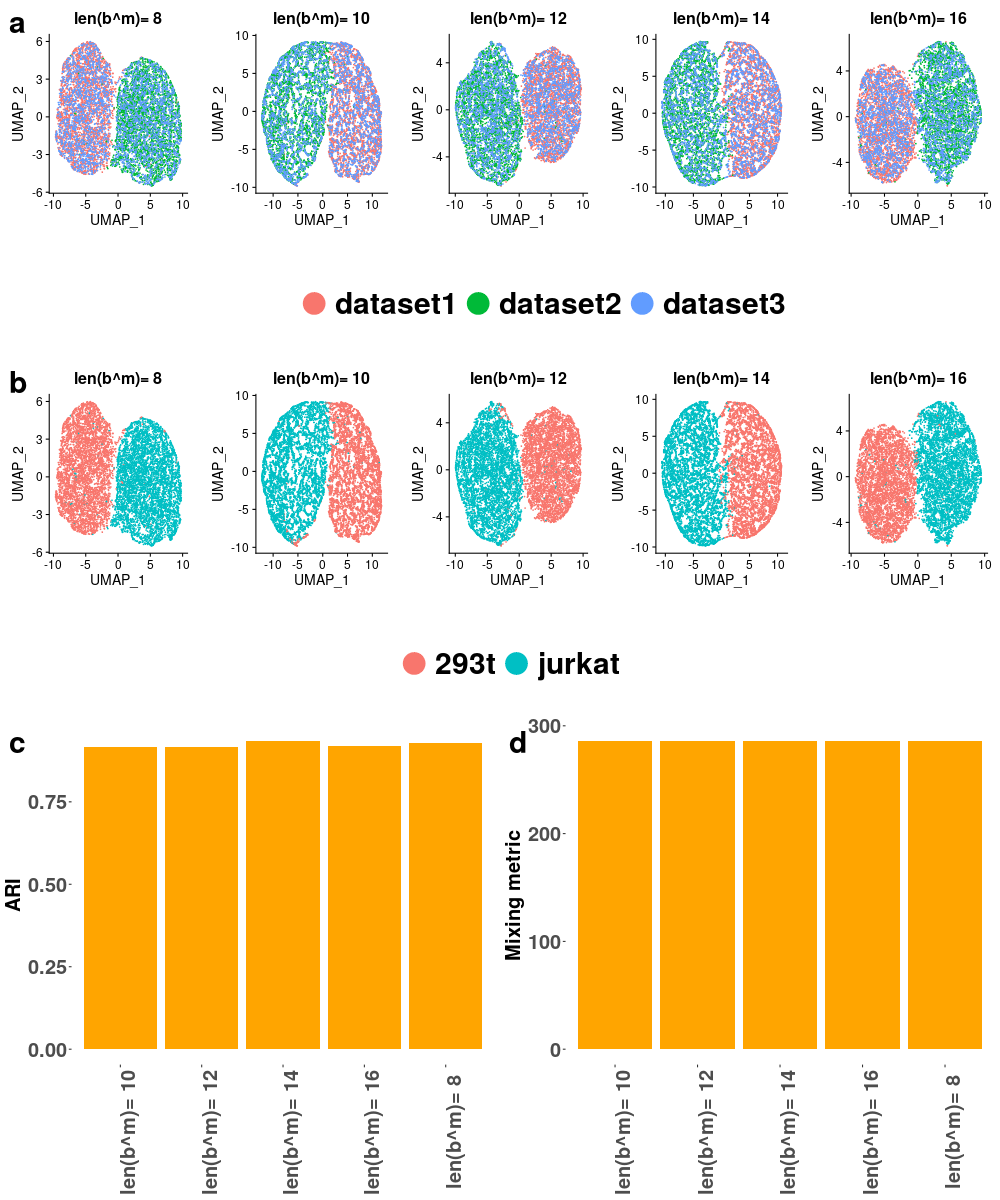


Figure S16 (a) UMAP visualization for the 293t and Jurkat cells from the three datasets in the second data application is shown across a range of dimensionality of _­_$b^{(m)}$ _._ Each dot represents a cell, colored by datasets (a) and by cell types (b). The stability of our method over a range of dimensionality of $b^{(m)}$ is measured by adjusted rand index (ARI) (c) and the mixing metric (d). ARI, mean=0.923, sd=0.0084; mixing metric, mean=286, sd=0.


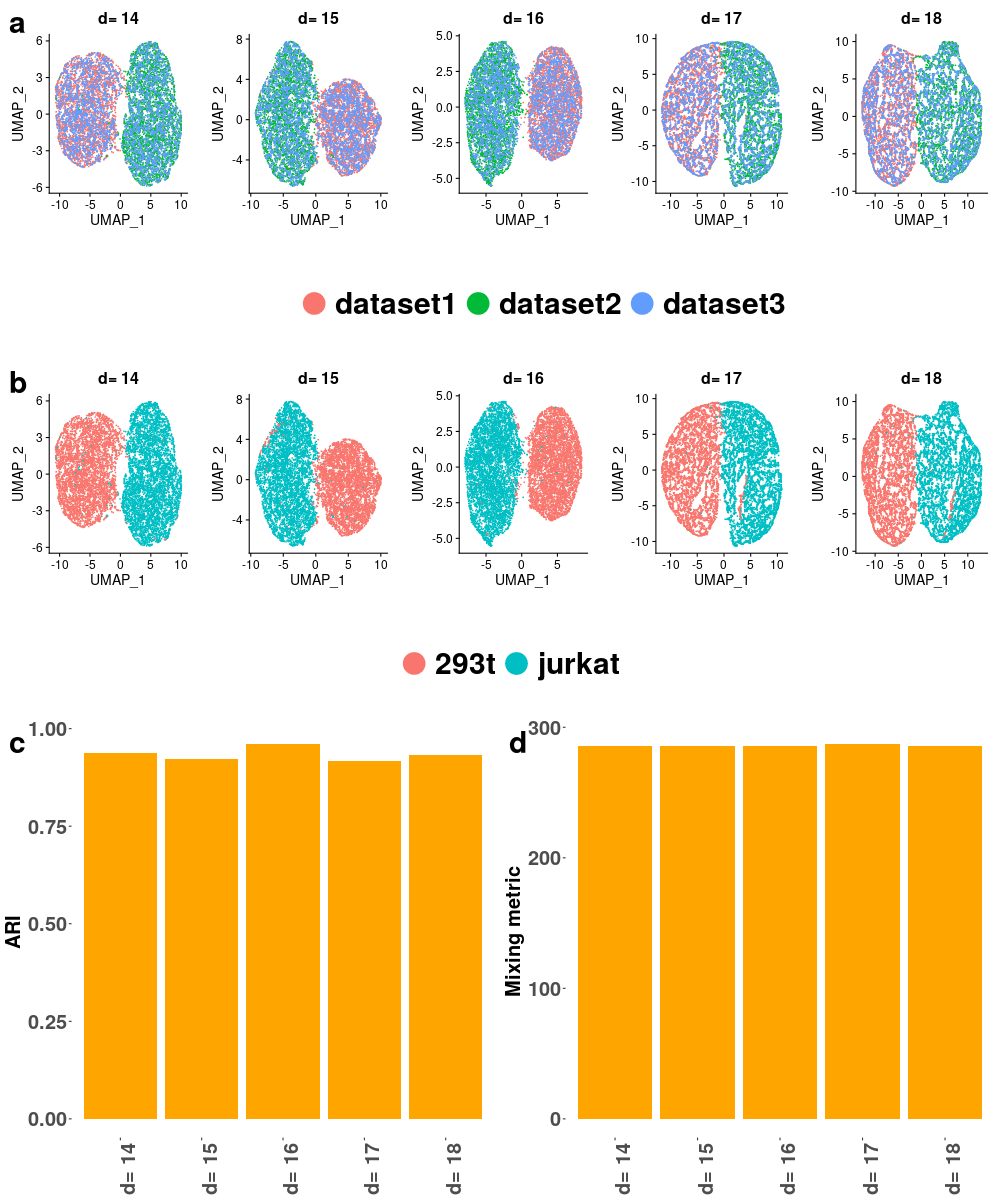


Figure S17 (a) UMAP visualization for the 293t and Jurkat cells from the three datasets in the second data application is shown across a range of dimensionality of the bottle layer_._ Each dot represents a cell, colored by datasets (a) and by cell types (b). The stability of our method over a range of dimensionality of the bottle layer is measured by adjusted rand index (ARI) (c) and the mixing metric (d). ARI, mean=0.933, sd=0.017; mixing metric, mean=286.2, sd=0.45.
