# Supplementary material for: Effective and scalable single-cell data alignment with non-linear canonical correlation analysis
Source: Nucleic Acids Res. 2021 Dec 6;50(4):e21. doi: 10.1093/nar/gkab1147 (PMC8887421; doi:10.1093/nar/gkab1147)
Supplement: gkab1147_Supplemental_Files [file gkab1147_supplemental_files.zip › Supplementary Text-20210804.docx]

# Supplementary Text

## Deep neural network in VIPCCA

We provide details for the deep neural network implemented in VIPCCA. The notations follow what has been described in the Materials and Methods section in the main text. In the neural network, the $l_{b}$-dimenisonal input layer $b^{\left( m \right)}$ is first transformed into another non-negative $l_{b}$-dimensional vector using two $l_{b}$-dimensional layers. The transformed vector is concatenated with $Z_{i}^{\left( m \right)}$ into a $(d+l_{b})$-dimensional layer. The resulting concatenated layer is connected to a *p*-dimensional layer through three intermediate layers with dimensionality 32, 64 and 128, respectively. We have another sub-branch which is connected from $Z_{i}^{\left( m \right)}$ to another $p$-dimensional layer through three intermediate layers with dimensionality 32, 64 and 128, respectively. The two $p$-dimensional layers are added to a final output $p$-dimensional layer. Overall, the two branches of the neural network is in the form of$\left\{ {d, l_{b}\to l}_{b}\to l_{b} \right\}\to\left( d+l_{b} \right)\to32\to64\to128\to p\to p$ and $d\to32\to64\to128\to p\to p$. The three $l_{b}$ layers inside the brace represent the input layer of $b^{\left( m \right)}$, the first intermediate layer with dimensionality $l_{b}$, and the second intermediate layer with dimensionality $l_{b}$, respectively. In the neural network, all inner layers except the two-inner p-dimensional layers (including the two intermediate $l_{b}$layers) are fully connected with each other through ReLU functions. The first inner $p$-dimensional layer is supplied with softplus as the activation function, the second inner $p$-dimensional layer with hard_sigmoid, the final output $p$-dimensional layer with ReLU. To ensure effective performance, we introduce a batch normalization layer before each ReLU activation layer and a dropout layer after the ReLU activation layer except the ReLU activation layer of the final output layer. Specifically, the batch normalization layer is designed to center and scale the inputs to a layer in a deep learning neural network, with the centering and scaling parameters treated as unknown and inferred through the inference algorithm. The batch normalization step can dramatically accelerate the inference of the neural network and in some cases can improve model performance through a modest regularization effect. In addition, the dropout layer is designed after the ReLU activation layer to randomly drop neuron units out (i.e. set these neuron output to be exactly 0) from the neural network during training with a droprate 1% in the present study. Notable, the dropout layers were only used for training the neural network, while the whole neural net was used without dropout at test time. To keep the expected summation over all outputs at test time is same as that at training time, the other 99% neurons values which were kept in the net are scaled up by 1/99% at training time. The dropout is a technique that can prevent overfitting and provide a way of approximating combining exponentially many different neural network architectures efficiently. For the dataset-specific input $b^{\left( m \right)}$, we simply generate a random $l_{b}$-dimensional integer vector from a discrete uniform distribution U(0, 10).

**Details for constructing the variational distributions**

To ensure flexible data modeling, we set the mean $\mu_{i}^{\left( m \right)}$ and variance $\sigma_{i}^{2\left( m \right)}$ in the variational distribution as functions of the observed expression data $X_{i}^{\left( m \right)}$. Because the posterior distribution for $Z_{i}^{\left( m \right)}$ is a non-linear function of the observed expression data $X_{i}^{\left( m \right)}$, we also use a non-linear function constructed by neural networks to characterize the mean and variance parameters in the variational distribution. By constructing non-linear functions of $X_{i}^{\left( m \right)}$ to represent $\mu_{i}^{\left( m \right)}$ and $\sigma_{i}^{2\left( m \right)}$, we hope to achieve a non-linear representation of the variational distribution and ensure the subsequent accurate approximation of the non-linear posterior distribution. Here, we follow the construction of $f(\cdot)$ and use neural network with a similar structure for $\mu_{i}^{\left( m \right)}$ and $\log\sigma_{i}^{2\left( m \right)}$. The neural network takes two inputs of the observed data $X_{i}^{\left( m \right)}$ and a dataset-specific integer vector $a^{\left( m \right)}$ and outputs two d-vector $\mu_{i}^{\left( m \right)}$ and $\log\sigma_{i}^{2\left( m \right)}$ through several intermediate neural network layers. Like the decoder, first, the input $l_{a}$-dimensional vector $a^{\left( m \right)}$ are transformed into another $l_{a}$-dimensional vector through two $l_{a}$-dimensional layers. In all our applications, we simply set $l_{a}=64$. Then, the transformed $l_{a}$-dimensional vectors and the input of $X_{i}^{\left( m \right)}$ are concatenated into a $\left( l_{a}+p \right)$-dimensional layer. The concatenated layer is connected to three layers with dimensionality of 128, 64 and 32, respectively. Finally, the 32-dimensional layer is connected to two d-dimensional output layers of $\mu_{i}^{\left( m \right)}$ and $\log\sigma_{i}^{2\left( m \right)}$. Just like the neural network in VIPCCA, inputs of each inner layer (i.e. layers except for the input, concatenated, and output layers) are fed to BatchNormalization, ReLU Activation, and Dropout layers (rate=0.01) as nonlinear configuration. The two output layers $\mu_{i}^{\left( m \right)}$ and $\log\sigma_{i}^{2\left( m \right)}$ are configured with two dense layers without an activation function (i.e. a linear combination of the previous layer). The latent representation $Z_{i}^{\left( m \right)}$ is randomly sampled by using a reparameterization technique $Z_{i}^{\left( m \right)}=\mu_{i}^{\left( m \right)}+\sigma_{i}^{\left( m \right)}\odot\epsilon, \epsilon\sim\mathcal{N}\left( 0,I \right)$. Note that we use $\mu_{i}^{\left( m \right)}$ as the final latent factors after we obtained the optimized neural network model, instead of using sampled $Z_{i}^{\left( m \right)}$.

## Variational inference algorithm

Our goal is to obtain estimates for $Z_{i}^{(m)}$, which represents the low-dimensional projects of the expression data collected from different datasets. Parameter estimation in our model through the maximum likelihood framework is not trivial, unfortunately, thanks to the nonlinear function $f(\cdot)$. Therefore, we instead develop a variational inference algorithm to enable efficient and effective parameter estimation. Specifically, the marginal log likelihood of the expression data, after integrating out of the latent variables $Z_{i}^{(m)}$, can be written as

$$\begin{aligned} \log p_{\theta}\left( X \right)=\Sigma_{m=1}^{k}\Sigma_{i=1}^{n_{m}}\log p_{\theta}\left( X_{i}^{\left( m \right)} | b^{\left( m \right)} \right)\#\#(1)\# \end{aligned}$$

with each term expressed as

$\begin{aligned} logp_{\theta}\left( X_{i}^{\left( m \right)}|b^{\left( m \right)} \right)=log\int p_{\theta}\left( X_{i}^{\left( m \right)}|Z_{i}^{\left( m \right)}, b^{\left( m \right)} \right)p\left( Z_{i}^{\left( m \right)} \right)dZ_{i}^{\left( m \right)}\#(2)\# \end{aligned}$ Above, the integrand is a product of two probabilities, with the first probability $p_{\theta}\left( X_{i}^{\left( m \right)}|Z_{i}^{\left( m \right)}, b^{\left( m \right)} \right)$ representing the likelihood and the second probability $p\left( Z_{i}^{\left( m \right)} \right)$ representing the prior. The integration in equation (2) has a closed form solution when $f\left( \cdot\right)$ is in a simple linear functional form as in PCCA. However, such integration is no longer available in an analytic form when we use the neural network to serve as the non-linear functional form of $f\left( \cdot\right)$.

With the variational distribution, we can re-express the log integration in equation (2) as

$\begin{aligned} log\int p_{\theta}\left( X_{i}^{\left( m \right)},Z_{i}^{\left( m \right)}|b^{\left( m \right)} \right)\frac{q\left( Z_{i}^{\left( m \right)} \right)}{q\left( Z_{i}^{\left( m \right)} \right)}dZ_{i}^{\left( m \right)}=\log\left( E_{q}\left[ \frac{p_{\theta}\left( X_{i}^{\left( m \right)},Z_{i}^{\left( m \right)}|b^{\left( m \right)} \right)}{q\left( Z_{i}^{\left( m \right)} \right)} \right] \right). \#\left( 3 \right) \end{aligned}$ In the above equation, the expectation $E_{q}$ is taken with respect to the variational distribution $q(Z_{i}^{\left( m \right)})$. With Jensen’s inequality, we further have

$$\log\left( E_{q}\left[ \frac{p_{\theta}\left( X_{i}^{\left( m \right)},Z_{i}^{\left( m \right)}|b^{\left( m \right)} \right)}{q\left( Z_{i}^{\left( m \right)} \right)} \right] \right)\geq E_{q}\left[ \log p_{\theta}\left( X_{i}^{\left( m \right)},Z_{i}^{\left( m \right)}|b^{\left( m \right)} \right) \right]-E_{q}\left[ \log q\left( Z_{i}^{\left( m \right)} \right) \right], \left( 4 \right)$$

where the right-hand side is the Evidence Lower Bound (ELBO). Our goal is to identify a variational distribution $q(Z_{i}^{\left( m \right)})$ that maximizes the ELBO, which equivalently minimizes the KL divergence between the variational distribution $q(Z_{i}^{\left( m \right)})$ and the posterior distribution $p\left( Z_{i}^{\left( m \right)}|X_{i}^{\left( m \right)}, b^{\left( m \right)},\theta,\sigma^{2} \right)$. To do so, we write the KL divergence between $q(Z_{i}^{\left( m \right)})$ and $p(Z_{i}^{\left( m \right)}\mid X_{i}^{\left( m \right)},b^{\left( m \right)},\theta,\sigma^{2})$ as:

$$KL(q(Z_{i}^{\left( m \right)})||p\left( Z_{i}^{\left( m \right)} | X_{i}^{\left( m \right)},b^{\left( m \right)},\theta,\sigma^{2} \right))=E_{q}\left[ \log\frac{q(Z_{i}^{\left( m \right)})}{p\left( Z_{i}^{\left( m \right)} | X_{i}^{\left( m \right)},b^{\left( m \right)},\theta,\sigma^{2} \right)} \right]$$

$$=E_{q}\left[ \log q\left( Z_{i}^{\left( m \right)} \right) \right]-E_{q}\left[ \log p\left( X_{i}^{\left( m \right)},Z_{i}^{\left( m \right)}|b^{\left( m \right)},\theta,\sigma^{2} \right) \right]+E_{q}\left[ \log p\left( X_{i}^{\left( m \right)}|b^{\left( m \right)},\theta,\sigma^{2} \right) \right]$$

$$\begin{aligned} =-\left( E_{q}\left[ \log p\left( X_{i}^{\left( m \right)},Z_{i}^{\left( m \right)}|b^{\left( m \right)},\theta,\sigma^{2} \right) \right]-E_{q}\left[ \log q\left( Z_{i}^{\left( m \right)} \right) \right] \right)+\log p\left( X_{i}^{\left( m \right)} | b^{\left( m \right)},\theta,\sigma^{2} \right)\#\left( 5 \right) \end{aligned}$$

In our variational neural network, the probabilistic encoder $q_{\varphi}(Z_{i}^{\left( m \right)}|X_{i}^{\left( m \right)},a^{\left( m \right)}$) is used as the variational distribution $q(Z_{i}^{\left( m \right)})$ in equation (5)**.** Hence, the marginal log-likelihood can be rewritten as:

$$\log p\left( X_{i}^{\left( m \right)} | b^{\left( m \right)},\theta,\sigma^{2} \right)=KL(q_{\varphi}\left( Z_{i}^{\left( m \right)} | X_{i}^{\left( m \right)},a^{\left( m \right)} \right)||p(Z_{i}^{\left( m \right)}|X_{i}^{\left( m \right)},b^{\left( m \right)},\theta,\sigma^{2}))+$$

$$\begin{aligned} \left( E_{q}\left[ \log p\left( X_{i}^{\left( m \right)},Z_{i}^{\left( m \right)}|b^{\left( m \right)},{\theta,\sigma}^{2} \right) \right]-E_{q}\left[ \log q_{\varphi}\left( Z_{i}^{\left( m \right)} | X_{i}^{\left( m \right)},a^{\left( m \right)} \right) \right] \right) \#\left( 6 \right) \end{aligned}$$

The first term and the second term in equation (6) are a KL regularizer and a ELBO. Since the KL divergence is non-negative, maximizing the ELBO is the same as minimizing the KL divergence from the variational distribution to the true posterior distribution $p\left( Z_{i}^{\left( m \right)} | X_{i}^{\left( m \right)},b^{\left( m \right)},\theta,\sigma^{2} \right)$, which is intractable. Specifically, the ELBO is:

$$\mathcal{L}\left( \theta,\varphi,\sigma;X_{i}^{\left( m \right)},b^{\left( m \right)},a^{\left( m \right)} \right)$$

$$\equiv E_{q}\left[ \log p\left( X_{i}^{\left( m \right)},Z_{i}^{\left( m \right)}|b^{\left( m \right)},{\theta,\sigma}^{2} \right) \right]-E_{q}\left[ \log q_{\varphi}\left( Z_{i}^{\left( m \right)} | X_{i}^{\left( m \right)},a^{\left( m \right)} \right) \right]$$

$$=E_{q}\left[ -\log q_{\varphi}\left( Z_{i}^{\left( m \right)} | X_{i}^{\left( m \right)},a^{\left( m \right)} \right)+\log p\left( X_{i}^{\left( m \right)},Z_{i}^{\left( m \right)}|b^{\left( m \right)},{\theta,\sigma}^{2} \right) \right]$$

$$=E_{q}\left[ -\log q_{\varphi}\left( Z_{i}^{\left( m \right)} | X_{i}^{\left( m \right)},a^{\left( m \right)} \right)+\log p\left( X_{i}^{\left( m \right)}|Z_{i}^{\left( m \right)},b^{\left( m \right)},\theta,\sigma^{2} \right)+\log p\left( Z_{i}^{\left( m \right)} | b^{\left( m \right)},\theta,\sigma^{2} \right) \right]$$

$$=E_{q}\left[ -\log\frac{q_{\varphi}\left( Z_{i}^{\left( m \right)} | X_{i}^{\left( m \right)},a^{\left( m \right)} \right)}{p\left( Z_{i}^{\left( m \right)} \right)} \right]+E_{q}\left[ \log p\left( X_{i}^{\left( m \right)}|Z_{i}^{\left( m \right)},b^{\left( m \right)},\theta,\sigma^{2} \right) \right]$$

$$=-KL(q_{\varphi}\left( Z_{i}^{\left( m \right)} | X_{i}^{\left( m \right)},a^{\left( m \right)} \right)||p\left( Z_{i}^{\left( m \right)} \right))+E_{q}\left[ \log p\left( X_{i}^{\left( m \right)}|Z_{i}^{\left( m \right)},b^{\left( m \right)},\theta,\sigma^{2} \right) \right] (7)$$

The above ELBO has two components. The first term is the KL divergence from the approximate posterior to the prior of $Z_{i}^{\left( m \right)}$, which acts as KL regularizer. The second term is an expected negative reconstruct error. However, the lower bound sometimes require adjustable pressure on reconstruction error when the KL regularizer may be too strong or too weak compared to the reconstruction loss. Hence, we used an adjusted constraint via $\lambda$ in the form:

$$\mathcal{L}\left( \theta,\varphi,\sigma;X_{i}^{\left( m \right)},a^{\left( m \right)},b^{\left( m \right)} \right)$$

$=-KL(q_{\varphi}\left( Z_{i}^{\left( m \right)} | X_{i}^{\left( m \right)},a^{\left( m \right)} \right)||p\left( Z_{i}^{\left( m \right)} \right))+\lambda E_{q}\left[ \log p\left( X_{i}^{\left( m \right)}|Z_{i}^{\left( m \right)},b^{\left( m \right)},\theta,\sigma^{2} \right) \right] \left( 8 \right)$.

We jointly learn the optimal parameter $\varphi^{*}$ and $\theta^{*}$ by maximizing the objective function across the k datasets as follows:

$$\varphi^{*},\theta^{*},\sigma^{*}={argmax}_{\varphi,\theta,\sigma}\sum_{m=1}^{k} \sum_{i=1}^{n_{m}} \{-KL(q_{\varphi}\left( Z_{i}^{\left( m \right)} | X_{i}^{\left( m \right)},a^{\left( m \right)} \right)||p\left( Z_{i}^{\left( m \right)} \right))+\lambda\cdot E_{q_{\varphi}\left( Z_{i}^{\left( m \right)} | X_{i}^{\left( m \right)},a^{\left( m \right)} \right)}\left[ \log p\left( X_{i}^{\left( m \right)}|Z_{i}^{\left( m \right)},b^{\left( m \right)},\theta,\sigma^{2} \right) \right]\} (9)$$

## Detailed optimization

In the lower bound $\mathcal{L}\left( \theta,\varphi,\sigma;X_{i}^{\left( m \right)},a^{\left( m \right)},b^{\left( m \right)} \right)$, the first term KL-divergence $KL(q_{\varphi}\left( Z_{i}^{\left( m \right)} | X_{i}^{\left( m \right)},a^{\left( m \right)} \right)||p\left( Z_{i}^{\left( m \right)} \right))$ can be computed and differentiated without estimation. To simplify our variational model, we assume the true posterior takes on a multivariate Gaussian with a diagonal covariance. Then the variational approximate posterior is in the form $q_{\varphi}\left( Z_{i}^{\left( m \right)} | X_{i}^{\left( m \right)},a^{\left( m \right)} \right)=\mathcal{N} (Z_{i}^{\left( m \right)};\mu_{i}^{\left( m \right)},diag(\sigma_{i1}^{2\left( m \right)},\sigma_{i2}^{2\left( m \right)},\ldots,\sigma_{\mathrm{id}}^{2\left( m \right)}))$. Let the prior distribution of $Z_{i}^{\left( m \right)}$ is also Gaussian $Z_{i}^{\left( m \right)}\mathcal{\sim N}(0,I)$, the KL divergence can be computed analytically as

$$-KL(q_{\varphi}\left( Z_{i}^{\left( m \right)} | X_{i}^{\left( m \right)},a^{\left( m \right)} \right)||p\left( Z_{i}^{\left( m \right)} \right))=-\int q_{\varphi}\left( Z_{i}^{\left( m \right)} | X_{i}^{\left( m \right)},a^{\left( m \right)} \right)\log\frac{q_{\varphi}\left( Z_{i}^{\left( m \right)} | X_{i}^{\left( m \right)},a^{\left( m \right)} \right)}{p\left( Z_{i}^{\left( m \right)} \right)}dZ_{i}^{\left( m \right)}$$

$$=-\int\{q_{\varphi}\left( Z_{i}^{\left( m \right)} | X_{i}^{\left( m \right)},a^{\left( m \right)} \right)\log q_{\varphi}\left( Z_{i}^{\left( m \right)} | X_{i}^{\left( m \right)},a^{\left( m \right)} \right)-q_{\varphi}\left( Z_{i}^{\left( m \right)} | X_{i}^{\left( m \right)},a^{\left( m \right)} \right)\log p(Z_{i}^{\left( m \right)})\}dZ_{i}^{\left( m \right)}$$

$$=-\frac{1}{2}\sum_{j=1}^{d} (\left( \mu_{ij}^{\left( m \right)} \right)^{2}+\left( \sigma_{ij}^{\left( m \right)} \right)^{2}-\log\left( \sigma_{ij}^{\left( m \right)} \right)^{2}-1)$$

Here, $\mu_{ij}^{\left( m \right)}$ and $\sigma_{ij}^{\left( m \right)}$ are the j-th element of the d-dimensional vector $\mu_{i}^{\left( m \right)}$ and $\sigma_{i}^{\left( m \right)}$, respectively. The second term of the lower bound $\mathcal{L}\left( \theta,\varphi,\sigma;X_{i}^{\left( m \right)},a^{\left( m \right)},b^{\left( m \right)} \right)$ is $E_{q}\left[ \log p\left( X_{i}^{\left( m \right)}|Z_{i}^{\left( m \right)},b^{\left( m \right)},\theta,\sigma^{2} \right) \right]$. For simplicity, let $f$,$\Sigma$ represent $f\left( Z_{i}^{\left( m \right)},b^{\left( m \right)}\mid\theta\right)$ and $diag(\sigma_{i1}^{2\left( m \right)},\sigma_{i2}^{2\left( m \right)},\ldots,\sigma_{id}^{2\left( m \right)})$, respectively. Then, the second term can be computed as follows:

$$E_{q}\left[ \log p\left( X_{i}^{\left( m \right)}|Z_{i}^{\left( m \right)},b^{\left( m \right)},\theta,\sigma^{2} \right) \right]$$

$$=\int\mathcal{N}\left( Z_{i}^{\left( m \right)}|\mu_{i}^{\left( m \right)},\Sigma\right)\log\mathcal{N}\left( X_{i}^{\left( m \right)}|f\left( Z_{i}^{\left( m \right)},b^{\left( m \right)}\mid\theta\right),\sigma^{2}I \right)dZ_{i}^{\left( m \right)}$$

$$=\int\frac{1}{\left( 2\pi\right)^{\frac{d}{2}}}\frac{1}{\mid\Sigma\mid^{\frac{1}{2}}}exp\left( -\frac{1}{2}\left( Z_{i}^{\left( m \right)}-\mu_{i}^{\left( m \right)} \right)^{T}\Sigma^{-1}\left( Z_{i}^{\left( m \right)}-\mu_{i}^{\left( m \right)} \right) \right)\log\left[ \frac{1}{\left( 2\pi\right)^{\frac{p}{2}}}\frac{1}{\mid\sigma^{2}I\mid^{\frac{1}{2}}}exp\left( -\frac{1}{2}\left( X_{i}^{\left( m \right)}-f \right)^{T}\left( \sigma^{2}I \right)^{-1}\left( X_{i}^{\left( m \right)}-f \right) \right) \right]dZ_{i}^{\left( m \right)}$$

$$=\int\left( 2\pi\right)^{-\frac{d}{2}}\prod_{j=1}^{d} \frac{1}{\sigma_{ij}^{\left( m \right)}}exp\left( -\frac{1}{2}\left( Z_{i}^{\left( m \right)}-\mu_{i}^{\left( m \right)} \right)^{T}\Sigma^{-1}\left( Z_{i}^{\left( m \right)}-\mu_{i}^{\left( m \right)} \right) \right)\left[ \left( -\frac{p}{2} \right)\log\left( 2\pi\right)+\left( -p \right)\log\sigma\right)+\left( -\frac{1}{2\sigma^{2}}\left( X_{i}^{\left( m \right)}-f \right)^{T}\left( X_{i}^{\left( m \right)}-f \right) \right)]dZ_{i}^{\left( m \right)}$$

$$=-\frac{p}{2}\left( \log\left( 2\pi\right)+\log\sigma^{2} \right)-\frac{1}{2}\int\left( 2\pi\right)^{-\frac{d}{2}}\prod_{j=1}^{d} \frac{1}{\sigma_{ij}^{\left( m \right)}}exp\left( -\frac{1}{2}\left( Z_{i}^{\left( m \right)}-\mu_{i}^{\left( m \right)} \right)^{T}\Sigma^{-1}\left( Z_{i}^{\left( m \right)}-\mu_{i}^{\left( m \right)} \right) \right)\frac{1}{\sigma^{2}}\left( X_{i}^{\left( m \right)}-f \right)^{T}\left( X_{i}^{\left( m \right)}-f \right)dZ_{i}^{\left( m \right)}$$

$$=-\frac{p}{2}\left( \log\left( 2\pi\right)+\log\sigma^{2} \right)-\frac{1}{2\sigma^{2}}\int\left( 2\pi\right)^{-\frac{d}{2}}\prod_{j=1}^{d} \frac{1}{\sigma_{ij}^{\left( m \right)}}exp\left( -\frac{1}{2}\left( Z_{i}^{\left( m \right)}-\mu_{i}^{\left( m \right)} \right)^{T}\Sigma^{-1}\left( Z_{i}^{\left( m \right)}-\mu_{i}^{\left( m \right)} \right) \right)\left( X_{i}^{\left( m \right)}-f \right)^{T}\left( X_{i}^{\left( m \right)}-f \right)dZ_{i}^{\left( m \right)}$$

$$=-\frac{p}{2}\left( \log\left( 2\pi\right)+\log\sigma^{2} \right)-\frac{1}{2\sigma^{2}}\int(\Sigma_{j=1}^{p}\left( X_{ij}^{\left( m \right)}-f_{j} \right)^{2})(\prod_{j=1}^{d} \frac{1}{{\sqrt{2\pi}\sigma}_{ij}^{\left( m \right)}})exp(-\frac{1}{2}\Sigma_{j=1}^{d}\frac{\left( Z_{ij}^{\left( m \right)}-\mu_{ij}^{\left( m \right)} \right)^{2}}{\sigma_{ij}^{2(m)}})dZ_{i}^{\left( m \right)}$$

As shown above, the expectation is not integrated analytically because of the intractable transformation function $f(\cdot)$. We use Monte Carlo estimator by sampling $Z_{i}^{\left( m \right)}$ $N$ times from $q_{\varphi}\left( Z_{i}^{\left( m \right)} | X_{i}^{\left( m \right)},a^{\left( m \right)} \right)$as follows:

$$E_{q_{\varphi}\left( Z_{i}^{\left( m \right)} | X_{i}^{\left( m \right)},a^{\left( m \right)} \right)}\left[ \log p\left( X_{i}^{\left( m \right)}|Z_{i}^{\left( m \right)},b^{\left( m \right)},\theta,\sigma^{2} \right) \right]\approx-\frac{p}{2}\left( \log\left( 2\pi\right)+\log\sigma^{2} \right)-\frac{1}{2{N\sigma}^{2}}(\Sigma_{l=1}^{N}\Sigma_{j=1}^{p}\left( X_{ij}^{\left( m \right)}-f_{j}\left( {Z_{i}^{\left( m \right)}}^{\left( l \right)},b^{\left( m \right)}|\theta\right) \right)^{2})$$

Let’s maximize this with respect to $\sigma$. If we take the derivative with respect to $\sigma$, we find that

$$\nabla_{\sigma}-\frac{p}{2}\left( \log\left( 2\pi\right)+\log\sigma^{2} \right)-\frac{1}{2{N\sigma}^{2}}\left( \Sigma_{l=1}^{N}\Sigma_{j=1}^{p}\left( X_{ij}^{\left( m \right)}-f_{j}\left( {Z_{i}^{\left( m \right)}}^{\left( l \right)},b^{\left( m \right)}|\theta\right) \right)^{2} \right)$$

$$=-\frac{p}{\sigma}+\frac{\sigma^{-3}}{N}\left( \Sigma_{l=1}^{N}\Sigma_{j=1}^{p}\left( X_{ij}^{\left( m \right)}-f_{j}\left( {Z_{i}^{\left( m \right)}}^{\left( l \right)},b^{\left( m \right)}|\theta\right) \right)^{2} \right)$$

Setting this to zero and solving for $\sigma^{2}$, it yields the update rule

$$\hat{\sigma^{2}}=\frac{1}{Np}\left( \Sigma_{l=1}^{N}\Sigma_{j=1}^{p}\left( X_{ij}^{\left( m \right)}-f_{j}\left( {Z_{i}^{\left( m \right)}}^{\left( l \right)},b^{\left( m \right)}|\theta\right) \right)^{2} \right)$$

Then, let’s maximize the expectation with respect to $\theta$. If we take the derivative with respect to $\theta$, we find that

$$\nabla_{\theta}-\frac{p}{2}\left( \log\left( 2\pi\right)+\log\sigma^{2} \right)-\frac{1}{2{N\sigma}^{2}}\left( \Sigma_{l=1}^{N}\Sigma_{j=1}^{p}\left( X_{ij}^{\left( m \right)}-f_{j}\left( {Z_{i}^{\left( m \right)}}^{\left( l \right)},b^{\left( m \right)}|\theta\right) \right)^{2} \right)$$

$$=-\frac{1}{2N\sigma^{2}}\left( \Sigma_{l=1}^{N}\Sigma_{j=1}^{p}\left( 2\left( X_{ij}^{\left( m \right)}-f_{j}\left( {Z_{i}^{\left( m \right)}}^{\left( l \right)},b^{\left( m \right)} \mid\theta\right) \right)\nabla_{\theta}f_{j}\left( {Z_{i}^{\left( m \right)}}^{\left( l \right)},b^{\left( m \right)} \mid\theta\right) \right) \right)$$

Setting this to zero, the update rule of $\theta$is

$$\Sigma_{l=1}^{N}\Sigma_{j=1}^{p}\left( \left( X_{ij}^{\left( m \right)}-f_{j}\left( {Z_{i}^{\left( m \right)}}^{\left( l \right)},b^{\left( m \right)} \mid\theta\right) \right)\nabla_{\theta}f_{j}\left( {Z_{i}^{\left( m \right)}}^{\left( l \right)},b^{\left( m \right)} \mid\theta\right) \right)=0$$

It yields an ELBO as follows:

$$\mathcal{L}\left( \theta,\varphi,\sigma;X_{i}^{\left( m \right)},a^{\left( m \right)},b^{\left( m \right)} \right)\approx-\frac{1}{2}\sum_{j=1}^{J} \left( \left( \mu_{ij}^{\left( m \right)} \right)^{2}+\left( \sigma_{ij}^{\left( m \right)} \right)^{2}-\log\left( \sigma_{ij}^{\left( m \right)} \right)^{2}-1 \right)-\lambda\cdot\frac{p}{2}\left( \log\left( 2\pi\right)+\log\sigma^{2} \right)-\frac{\lambda}{2{N\sigma}^{2}}\left( \Sigma_{l=1}^{N}\Sigma_{j=1}^{p}\left( X_{ij}^{\left( m \right)}-f_{j}\left( {Z_{i}^{\left( m \right)}}^{\left( l \right)},b^{\left( m \right)}\mid\theta\right) \right)^{2} \right)$$

$$Z_{i}^{\left( m \right)}\mathcal{\sim N(}\mu_{i}^{\left( m \right)},{diag(\sigma}_{i1}^{2\left( m \right)},\sigma_{i2}^{2\left( m \right)},\ldots,\sigma_{id}^{2\left( m \right)}))$$

We rely on neural network to learn the parameters $\mu_{i}^{\left( m \right)}$ and $\sigma_{i}^{\left( m \right)}$. However, a directly sampling of $Z_{i}^{\left( m \right)}$ from $q_{\varphi}\left( Z_{i}^{\left( m \right)} | X_{i}^{\left( m \right)},a^{\left( m \right)} \right)$ would result in indifferentiable bottleneck layers in the neural networks, which hinder the backpropagation to update $\mu_{i}^{\left( m \right)}$ and $\sigma_{i}^{\left( m \right)}$. To make the two parameters be learnable in neural networks, we need to implement the sampling function with a neural network such that the function $Z_{i}^{\left( m \right)}=f_{sampling}(\mu_{i}^{\left( m \right)},\sigma_{i}^{\left( m \right)})$ is differentiable with respect to $\mu_{i}^{\left( m \right)}$ and $\sigma_{i}^{\left( m \right)}$. To solve this problem, we used the reparameterization trick $Z_{i}^{\left( m \right)}=\mu_{i}^{\left( m \right)}+\sigma_{i}^{\left( m \right)}\odot\epsilon, \epsilon\sim\mathcal{N}(0,I)$, which is to rewrite the expectation so that the distribution $p\left( \epsilon\right)$ we take samples from is independent from the parameters $\mu_{i}^{\left( m \right)}$ and $\sigma_{i}^{\left( m \right)}$ we are aiming to learn. Here, $\odot$ represents element-wise multiplication of two vectors. Thus, our estimator of the ELBO can be rewritten as follows:

$$\tilde{\mathcal{L}}\left( \theta,\varphi,\sigma;X_{i}^{\left( m \right)},a^{\left( m \right)},b^{\left( m \right)} \right)=-\frac{1}{2}\sum_{j=1}^{J} \left( \left( \mu_{ij}^{\left( m \right)} \right)^{2}+\left( \sigma_{ij}^{\left( m \right)} \right)^{2}-\log\left( \sigma_{ij}^{\left( m \right)} \right)^{2}-1 \right)-\lambda\cdot\frac{p}{2}\left( \log\left( 2\pi\right)+\log\sigma^{2} \right)-\frac{\lambda}{2{N\sigma}^{2}}\left( \Sigma_{l=1}^{N}\Sigma_{j=1}^{p}\left( X_{ij}^{\left( m \right)}-f_{j}\left( \mu_{i}^{\left( m \right)}+\sigma_{i}^{\left( m \right)}\odot\epsilon^{\left( l \right)},b^{\left( m \right)}\mid\theta\right) \right)^{2} \right)$$

$$\epsilon\sim\mathcal{N}(0,I)$$

The objective function across the whole datasets is in the form as follows:

$$\begin{aligned} \varphi^{*},\theta^{*},\sigma^{*}=argmax_{\varphi,\theta,\sigma} \Sigma_{m=1}^{k}\Sigma_{i=1}^{n_{m}}\tilde{\mathcal{L}}\left( \theta,\varphi,\sigma;X,a^{\left( m \right)},b^{\left( m \right)} \right)\#\left( 10 \right) \end{aligned}$$

## Other related methods to VIPCCA

Our deep nonlinear multi-modal generative probabilistic model specified in Material and Methods is closely related to several statistical models that include the probabilistic CCA, the multimodal factor analysis, the inter-battery factor analysis, the deep CCA, and the variational CCA.

**Canonical correlation analysis**

Canonical correlation analysis (CCA) [1] is a classical method for seeking correlations between two multivariate data sets, which was widely used as initial dimensional reduction to find matching correspondences across a paired datasets. Mathematically, given two multivariate random variables with n observations as matrices $X^{\left( 1 \right)}\in R^{m_{1}\times n}$ and $X^{\left( 2 \right)}\in R^{m_{2}\times n}$, CCA is aiming to find a pair of linear transformation $U\in R^{m_{1}\times d}$ and $V\in R^{m_{2}\times d}$ so that the correlation between $u_{k}^{T}X^{\left( 1 \right)}$ and $v_{k}^{T}X^{\left( 2 \right)}$ is maximized for the components k, under the constraint that $u_{k}^{T}X^{\left( 1 \right)}$ and $u_{k'}^{T}X^{\left( 1 \right)}$ are uncorrelated for all $k\neq k'$ (and similarly for $X^{\left( 2 \right)}$).

**Probabilistic CCA**

It assumes that $x_{1}$ and $x_{2}$ depend on a common latent random vector $z$ in a reduced dimensional space. CCA can also be treated as a linear probabilistic model of two random multivariate variables $x_{1}$ and $x_{2}$ with a latent variable $z\in R^{d\times1}$ [2]. The model is formulated as:

$$z\sim\mathcal{N}\left( 0,I_{d} \right),$$

$$x^{\left( 1 \right)}|z\sim\mathcal{N}\left( W_{1}z+u_{1},\Psi_{1} \right), W_{1}\in R^{m_{1}\times d},\Psi_{1}\geq0$$

$$x^{\left( 2 \right)}|z\sim\mathcal{N}\left( W_{2}z+u_{2},\Psi_{2} \right),W_{2}\in R^{m_{2}\times d},\Psi_{2}\geq0$$

$\mathrm{where}d\leq min\{m_{1},m_{2}\}$. It proves that the maximum likelihood estimates of the probabilistic model lead to the canonical correlation directions. As a generalization of the probabilistic CCA (PCCA), our model proposed in the present studies used a nonlinear function $f(\cdot)$ and a dataset-specific vector $b^{\left( m \right)}$ to transform the shared latent variable $z$ into multiple observation spaces of multiple multivariate random variables $x^{\left( 1 \right)}{,x}^{\left( 2 \right)},\ldots,x^{(k)}$. In particularly, our model will be equivalent to the PCCA model based on an assumption that the function $f\left( \cdot\right)$ is linear and the residual error has a dataset-specific generic covariance $\Psi^{\left( m \right)}$.

**Inter-battery factor analysis**

In statistical literature, it is also commonly known as the inter battery factor analysis (IBFA) is a generalization of canonical correspondence analysis (CCA) into multi-view data analysis [3]. Let $x^{\left( 1 \right)}\in R^{p_{1}\times1},x^{\left( 2 \right)}\in R^{p_{2}\times1},\ldots,x^{\left( k \right)}\in R^{p_{k}\times1}$ are k multivariate random variables and $X^{\left( 1 \right)},X^{\left( 2 \right)},\ldots,X^{\left( k \right)}$ are $n_{1},n_{2},\ldots,n_{k}$ i.i.d. observations from the k variables, respectively. The original IBFA model was defined as:

$$z\sim\mathcal{N}(0,\Phi)$$

$$z^{\left( m \right)}\mathcal{\sim N}(0,\Theta)$$

$$e^{\left( m \right)}\mathcal{\sim N}(0,\Psi)$$

$$x^{\left( m \right)}=\mu^{\left( m \right)}+\Lambda^{\left( m \right)}z+\Gamma^{\left( m \right)}z^{\left( m \right)}+e^{\left( m \right)},m\in\{1,2,\ldots,k\}$$

In this model, it represents the inter-battery factors by $z$, battery-specific factors by $z^{\left( m \right)}$. According to the probabilistic interpretation of IBFA [4, 5], the models can also be written as follows:

$$z\sim\mathcal{N}\left( 0,I \right)$$

$$z^{\left( m \right)}\mathcal{\sim N}\left( 0,I \right)$$

$$x^{\left( m \right)}\mathcal{\sim N}\left( A^{\left( m \right)}z+B^{\left( m \right)}z^{\left( m \right)},\Sigma^{\left( m \right)} \right),m\in\{1,2,\ldots,k\}$$

In the probabilistic IBFA, the shared latent variable $z\in R^{d\times1}$ (in lower dimensional space) captures variation common to all k datasets, and they are transformed to the observation space by the linear mappings $A^{\left( m \right)}z$, where $A^{\left( m \right)}\in R^{p_{m}\times d}$. The remaining variation is modelled by the latent variables $z^{\left( m \right)}\in R^{d_{m}\times1}$ specific to each dataset, transformed to the observation space by another linear mapping $B^{\left( m \right)}z^{\left( m \right)}$, where $B^{\left( m \right)}\in R^{{p_{m}\times d}_{m}}$. In the model, $\Sigma^{m}$ are diagonal matrices, indicating independence of the noise over the features. This probabilistic model can be converted into the probabilistic CCA by integrating out the dataset-specific latent variable $z^{\left( m \right)}$[2]. It can be formulated as:

$$z\sim\mathcal{N}(0,I)$$

$$x^{\left( m \right)}\mathcal{\sim N(}A^{\left( m \right)}z,\Psi^{\left( m \right)}), m\in\{1,2,\ldots,k\}$$

where $z$ is the only latent variable, and $\Psi^{\left( m \right)}$is a generic covariance matrix. It proved that this model is equivalent to classic CCA [4].

**Deep CCA**

An existing non-linear extension of CCA, called deep CCA (DCCA) [6], was designed to find a pair of deep neural networks to learn non-linear transformation of two datasets to a lower dimensional space in which the data is highly correlated. However, it does not provide a model for generating samples from the latent space. It computes representation of the two datasets by jointly learning parameters for two separate deep neural networks, which cannot be directly used for multiple datasets. Its canonical correlation loading is not necessarily aligned because of potential shift of feature space and population density.

**Deep generative CCA**

A deep variational CCA (VCCA) [7] extends the probabilistic linear CCA to a non-linear multi-view model parameterized by deep neural networks. Given two views $x^{\left( 1 \right)}$ and $x^{\left( 2 \right)}$, it attempts to learn non-linear models $p_{\theta}(x^{\left( 1 \right)}|z;\theta_{x})$ and $p_{\theta}\left( x^{\left( 2 \right)} | z;\theta_{y} \right)$ parametrized with $\theta_{x}$ and $\theta_{y}$, which was learned by using two separate neural networks. VCCA assumes the common latent variables $z$ is sufficient to generate both of the two views, which can be too restrictive in practice. Thus, a variant of VCCA was proposed with two additional sets of hidden variables $h_{x}$ and $h_{y}$ to explain view-specific variations not captured by the common variable $z$. However, it costs more computational resources in learning neural network parameters to infer additional hidden variables.

**Distinguished features of our model**

Our deep nonlinear PCCA model distinguishes itself from deepCCA and VCCA by using a probabilistic non-linear model with a shared inter-battery, latent variable parameterized by shared deep neural networks. It avoids training condition-specific neural networks to learn the distribution of expression data. And it was specially designed for the integration of multiple scRNA-seq datasets. Additionally, cell representation in the reduced dimensional space don’t require further normalization or adjustment, because its shared latent variable reflects the common variation across all datasets. Thus, we take advantage of deep neural networks to jointly learn a variational inference model and a generalized PCCA model to identify the shared latent inter-battery factor $z$, which could represent the common variation of all datasets. Our model and inference algorithm is also closely related to the conditional variational auto encoder (CVAE) [8].

**References**

1. HOTELLING, H., *RELATIONS BETWEEN TWO SETS OF VARIATES*.* Biometrika, 1936. **28**(3-4): p. 321-377.

2. Bach, F.R. and M.I. Jordan, *A probabilistic interpretation of canonical correlation analysis.* 2005.

3. Tucker, L.R., *An Inter-Battery Method of Factor-Analysis.* Psychometrika, 1958. **23**(2): p. 111-136.

4. Browne, M.W., *MAXIMUM-LIKELIHOOD SOLUTION IN INTER-BATTERY FACTOR-ANALYSIS.* British Journal of Mathematical & Statistical Psychology, 1979. **32**(MAY): p. 75-86.

5. Klami, A., S. Virtanen, and S. Kaski, *Bayesian Canonical Correlation Analysis.* Journal of Machine Learning Research, 2013. **14**: p. 965-1003.

6. Andrew, G., et al. *Deep canonical correlation analysis*. in *International conference on machine learning*. 2013.

7. Wang, W., et al., *Deep variational canonical correlation analysis.* arXiv preprint arXiv:1610.03454, 2016.

8. Klys, J., J. Snell, and R. Zemel. *Learning latent subspaces in variational autoencoders*. in *Advances in Neural Information Processing Systems*. 2018.
